# Improvement of Hexacopter UAVs Attitude Parameters Employing Control and Decision Support Systems

**DOI:** 10.3390/s23031446

**Published:** 2023-01-28

**Authors:** Mihai-Alin Stamate, Cristina Pupăză, Florin-Adrian Nicolescu, Cristian-Emil Moldoveanu

**Affiliations:** 1 Faculty of Industrial Engineering and Robotics, University Politehnica of Bucharest, 060042 Bucharest, Romania; 2 Faculty of Integrated Weapon Systems, Military Technical Academy “Ferdinand I”, 050141 Bucharest, Romania

**Keywords:** sensor systems, remote control and communication, UAV, simulation

## Abstract

Today, there is a conspicuous upward trend for the development of unmanned aerial vehicles (UAVs), especially in the field of multirotor drones. Their advantages over fixed-wing aircrafts are that they can hover, which allows their usage in a wide range of remote surveillance applications: industrial, strategic, governmental, public and homeland security. Moreover, because the component market for this type of vehicles is in continuous growth, new concepts have emerged to improve the stability and reliability of the multicopters, but efficient solutions with reduced costs are still expected. This work is focused on hexacopter UAV tests carried out on an original platform both within laboratory and on unrestricted open areas during the start–stop manoeuvres of the motors to verify the operational parameters, hover flight, the drone stability and reliability, as well as the aerodynamics and robustness at different wind speeds. The flight parameters extracted from the sensor systems’ comprising accelerometers, gyroscopes, magnetometers, barometers, GPS antenna and EO/IR cameras were analysed, and adjustments were performed accordingly, when needed. An FEM simulation approach allowed an additional decision support platform that expanded the experiments in the virtual environment. Finally, practical conclusions were drawn to enhance the hexacopter UAV stability, reliability and manoeuvrability.

## 1. Introduction

DRONE is a generic name for a whole family of aerial, land, water, and underwater platforms. The term DRONE is an English acronym, one of the definitions being Dynamic Remotely Operated Navigation Equipment. The following main categories of vehicles belong to the DRONE family are as follows: UAV—unmanned aerial vehicle, UGV—unmanned ground vehicle and UUV—unmanned underwater vehicle. 

Aerial drones are also found under other names: UAV—unmanned aerial vehicle, UAS—unmanned aerial system, RPAS—remotely piloted aircraft system and ROAV—remotely operated air vehicle.

UAVs fall into two main categories: fixed wing (airplane) and rotorcraft (single rotor—helicopter, or at least two rotors—multicopters). Recently (2020–2022), a third category of UAVs has seen rapid development: fixed-wing UAVs with vertical takeoff and landing (VTOL) capabilities [1,2,3], which combine the capabilities of an aircraft with those of a multirotor UAV, with either electric or combined propulsion [4,5,6] (electric with internal combustion engine) to extend flight range and develop superior flight performance, with the aim of being able to carry large payloads over long distances.

The main purpose for which UAVs were originally developed was their use in military applications and special operations. Subsequently, they have been widely developed and employed in an increasing number of civilian applications: law enforcement surveillance missions, firefighting assistance, securing borders, strategic and governmental targets, detecting illegal hunting, measuring landslides, monitoring incidents involving crowds of people, inspecting large industrial facilities, large buildings and constructions, oil and gas pipelines, inspection of continuous-flow machinery in quarries (to monitor temperatures in the area of high-friction pits using thermal imaging cameras), inspection of petrochemical installations (to detect cracks, fissures and leaks in pressure vessels using thermal imaging cameras) and, more recently, (2020–2022) home parcel delivery [7,8], warehouse stock management [9,10] using specialized software, passenger transport, air travel, etc.

Multirotor drones have seen continuous development over the past ten years as the need for this type of platform has grown continuously, and they are employed in a wide range of activities and fields such as inspection of large industrial installations [11,12], large buildings and constructions, oil and gas pipelines [13,14], inspection of continuous-flow machinery in quarries (to monitor temperatures in the area of high-friction pits using thermal imaging cameras), inspection of petrochemical installations (to detect cracks, fissures and leaks that may occur in pressure vessels using thermal imaging cameras [15,16]), etc. They can be equipped with a wide range of electromagnetic spectrum sensors [17,18], gamma ray sensors [19], biological sensors [20,21,22] and chemical sensors [23,24], which provide remote sensing functions.

Electromagnetic sensors include visual spectrum, infrared or near-infrared cameras and radar systems. Some other electromagnetic wave detectors such as microwave and ultraviolet spectrum sensors are less used. Biological sensors can detect the presence of various microorganisms and other biological factors in the air. Chemical sensors employ laser spectroscopy to analyse the concentration of each element in the air.

A UAV possesses almost all the characteristic strengths of a manned aircraft, in addition to surmounting the physical limitations of the pilots and thus preventing the human error. The absence of the pilot from the cockpit allows drones to be operated at their performance limit, thus increasing endurance, payload, altitude ceiling and manoeuvrability. 

Likewise, current advances in microelectronics and proximity/optical sensors [25,26], coupled with the availability of detailed geographic information systems mapping [27,28,29], contributed to the development of micro-UAVs [30] that can be operated autonomously at very low altitudes within dense urban locations and provide accurate intelligence data.

This paper aims to treat the category of multicopters UAVs, specifically the category of hexacopter drones. Given the upward trend in the aviation industry in the field of unmanned aerial vehicles (UAVs), especially in the field of multirotor drones [31,32,33,34], whose advantage over fixed-wing aircraft (airplanes) is that they can hover at a fixed point, which obviously allows their use in remote surveillance applications of different types of targets, the use of UAVs in the field of surveillance is also a key issue, and, at the same time, taking into account that the market for components for this type of vehicle is constantly growing and developing, with increasingly lower costs. Due to their cost efficiency and numerous possibilities for use in a wide range of civil, commercial and industrial applications (inspection of power lines, inspection of road infrastructure, bridges, inspection of oil pipelines, inspection of industrial facilities of strategic interest, e.g., oil refineries, nuclear power plants, inspection of disaster areas), multirotor UAVs have already been the subject of study for more than a decade. Since then, numerous research studies have been carried out on the modelling [33,34,35] and design of actuation, command and control systems [36] and the development of various design solutions [37,38]. Despite the abundance of the scientific literature, the topicality of the subject remains high, due to the subtle balance between the sensor features and involved outlays.

This paper is structured as follows: Section 2 encompasses a synthetic and critical overview of the latest developments in hexacopter drones design, sensors equipment and experimental procedures but also regarding numerical evaluation of the drone stability and reliability. Section 3 depicts a novel hexacopter platform architecture in two variants, equipped with avionic components and sensors. In Section 4, the results of the carried-out tests, both in the laboratory and in situ, during the start–stop manoeuvres of the hexacopter engines are described and discussed, and it also discusses the necessary corrective measures that were taken accordingly to check and assure the reliability of the hexacopter. Section 5 encompasses an extended FEM decision support study to investigate if resonances of the structural components interfere with the operational frequencies in order to avoid flight instabilities. The structural integrity of the hexacopter in case of a drop event was assessed, and the influence of the air pressure at different wind speeds was also investigated in respect to the hexacopter flight path accuracy. The accomplishments are summarized in Section 6, where conclusions are drawn and perspectives for the future work are highlighted.

The novelty of the work consists of a new perspective of comparative analyses of the performance of the hexacopter drone in different equipment variants in terms of battery, propellers, avionics components and engines employed by carrying out simulations using devoted simulation platforms. These studies also allowed us to establish the appropriate criteria for choosing the best combination of the propulsion system, consisting of a battery, such as an electronic speed controller (ESC), or a brushless DC motor (BLDC)—propeller—depending on the size of the drone frame, in order to achieve maximum efficiency (range vs. maximum carried payload).

Another original viewpoint covered in the article is the extended CFD study of the behaviour of the hexacopter drone during stationary flight at a fixed point (hover), which deals with several aspects, namely, ensuring the stability of the hexacopter drone during stationary flight manoeuvres at hover, development of a complete and complex simulation model for all types of CAE analysis, validation of the FEM (finite element model) computation model, synchronization of the results obtained analytically, experimentally and numerically and the use of the results obtained from the FEM study to improve flight parameters such as rotor speeds.

## 2. Related Work

### 2.1. Latest Developments in Hexacopter Drones Design

Darvishpoor et al. [39] present in a complex review many different configurations, flight mechanisms and applications in which drones are currently employed. The UAVs are categorized, and their characteristics, advantages and drawbacks are discussed. This study also presents vertical takeoff and landing (VTOL) hexacopter drones in a flat configuration, used by the last mile delivery drone, the HexH2O seaplane drone, an antidrone hexacopter, which uses a net to capture rogue drones; power tower cleaning hexacopters; and agriculture, inspection, survey and mapping hexacopters.

Delbecq et al. [40] presents a generic methodology that analyses the sizing aspect of the multicopter drones with electric propulsion, which allows configuration optimization for different applications. The study starts from a set of algebraic equations based on scaling laws and models that have resemblances. In the next phase, the optimization of the drone sizing is analysed through a proposed methodology. The obtained results are validated by comparing the characteristics of existing multirotors and performance predictions of these configurations which were performed taking into account different flight types and payload variants.

In the case of the classic hexacopter, studies have been carried out on mounting the rotors under certain tilt angles, this modification allowing the hexacopter to be fully actuated in the sense that all six degrees of freedom associated with the three translational and three rotational movements become independently controllable. These types of platforms are still the subject of study, making it difficult to explain which type of structure is suitable for a particular type of application. One of the proposed approaches to obtain a structure close to the one already mentioned is to develop a scheme to optimize the construction design of the drone. Aspects related to the design and optimization of hexacopter drones can be found in several variants proposed by Gupta et al. [41], Suprapto et al. [42], Setiono et al. [43], Verbeke et al. [44], Abarca et al. [45] and Arellano-Quintana et al. [46].

Work performed by Ferrarese et al. [47], Ryll et al. [48] and Tadokoro et al. [49], respectively, present an analytical characterization of the relationships between the dynamic properties of the drone, the arrangement of the rotors and their pitch angles. The results obtained are then taken into account when formulating the design aspects of the hexacopter and the manoeuvrability of the fully powered drone is analysed after the rotors are placed under a certain tilt angle. The following aspects are addressed: the effect of the rotor placement is analysed using the dynamic model of the hexacopter and the dynamic manipulability measure (DMM)—an index measuring the omnidirectional acceleration. An adapted version of the DMM suitable for a hexacopter is introduced. The DMM evaluates the input–output relationship between the thrust developed by the rotors and the acceleration of the vehicle and the introduction of a new type of structure, namely, symmetric coplanar tilted rotor (SCTR). The DMM method applied to the hexacopter in the new SCTR structure is considered suitable for the evaluation of the integral drive property of the multirotor structure. Finally, issues of optimizing the hexacopter construction design are considered by Rajappa et al. [50].

Köse et al. [51] presented an interesting approach that combines modelling and simulation for different drone configurations, employing an original software combination between Solidworks–PID Simulink and SPSA (Simultaneous Perturbation Stochastic Approximation) to develop an algorithm for optimizing flight parameters and studying different flight regimes for a certain fixed length of the motor support arms.

Mehmood et al. [52] depict the manoeuvrability of a fully powered hexacopter over all six degrees of freedom by installing all the propellers under the same pitch angle. In order to evaluate the manoeuvrability, a biaxial propeller tilt was considered to allow for two possible study situations, i.e., inward and/or lateral propeller tilt. Over a wide range of tilt angles, it was discovered, for all six degrees of freedom, that inward tilt of the propellers either results in decreased drone manoeuvrability or provides less optimal gains at a low cost of efficiency of the propulsion system.

Budinger et al. [53] present several models to estimate the performances of the main components of multirotor drones with electric propulsion. The mathematical models described in the paper facilitate the employment of design and optimisation tools. Using the current available technologies, these models can be employed for the preliminary design of new sensor systems. Alternate developing methods were utilized to find an analytical model built on datasheet records (propellers) and on FEM simulations records (landing gear). Thus, the dimensional assessment simplifies the selection of the primary individual parameters and increases the assessment of the models.

The present paper comprises an original design and deployment of the hexacopter, the extended experimental study, the choice and integration of avionics components, command and control, video acquisition, telemetry data and the explanations regarding the future development of an equipment variant for the command and control of the hexacopter out of direct line of sight (BVLOS). 

### 2.2. Sensors Equipment 

In addition to the propulsion system, the sensors with which the drone is equipped play a critical role in terms of manoeuvrability, stability, command and control of the drone. The sensors also capture information from the surrounding environment (images, video, GPS location, photogrammetry, LIDAR), depending on the specific missions or activities that the drone is meant to perform. 

Hussein and Nouacer [54] provide a source design pattern for building new drone systems, which includes blocks of the drones and relations between them that are distributed into four main groups: flight navigation, flight control, flight management and mission supervision.

Cao et al. [55] treat the examination of low power transmission lines using multicopter drones, in terms of making the right decision, cantered on data fusion acquired from a multisensor system. The information fused refers to the main aspects affecting the UAV parameters (flight speed), wind velocity, errors of the navigation positioning and size of the drone frame. A method called MFD-LPTL (multisensor fusion data analysis for low power transmission lines) is presented. This method’s main purpose is to conceive a model for secure distance prediction between the drone and the power lines. Based on the multisensor data fusion, combining data from various sensors including radars, LIDAR and camera models, a statistical model which uses the autonomous avoidance navigation of the problems was applied. 

Severin and Soffker [56] treat the problem of optimization of the sensors used for altitude estimation mounted on multicopter drones employed for spraying the vineyards. The study makes a comparison between a variety of low-cost sensors for measuring the distance between the drone and ground level, sensors which are most appropriate for vineyard-spraying drones. The signals were acquired from ultrasound, radar and Doppler sensors and were filtered using a Kalman filter. The study describes a variety of measures employed to improve the assessed altitude of the drone and to enhance the trustworthiness regarding the relative altitude approximation of the multicopter drone. 

In Pena et al.’s [57] WILD HOPPER UAV study, a 600 *L* platform designed for forest firefighting is presented. The paper reveals a multilayer steadiness system for enhanced stability of the drone during the flight in severe conditions. WILD HOPPER is equipped with a range of sensors which include thermal cameras, geolocalization and navigation systems: satellite navigation and a new technology based on visual attitude estimation methods. 

The study presented by Ravin et al. [58] explains the extraction and analysis of GPS data from three different drone manufacturers, followed by analysis and representation of the positioning data as flight paths. GPS-related data from any drone’s flight is of vital importance as it helps in establishing a legal framework for operating a drone in a country’s airspace. In terms of sensors, all these data are obtained from the GPS antenna/antennae mounted on the drone. 

To be able to fly, a multicopter drone needs a flight controller, which is the brain of the drone. In terms of sensors, this flight controller consists of an AHRS (attitude and heading reference system) IMU (inertial measurement unit), which is a device that integrates multiaxes accelerometers, gyroscopes and magnetometers to provide estimation of the drone’s orientation in space, providing measurements of pitch, roll and yaw. When the drone flies in an environment where GPS signal can be acquired, to ensure the reliability and highest performance, a sensor that includes an AHRS, as well as a GNSS receiver, which utilizes the GPS, GLONASS, BeiDou and Galileo satellite constellations, provide the best navigation system.

If the drone needs to be flown BVLOS (beyond visual line-of-sight) without being reliant on GPS, it is mandatory to have mounted on the drone a fully calibrated and temperature compensated AHRS IMU sensor under all dynamic conditions. In order to compensate for the three-axis movement of the drone based on user’s input, the flight controller needs PID (proportional-integral-derivative) controllers or combinations of these (PI, PD and so on).

The study performed by Sree Ezhil et al. [59] concentrates on the efficacy of PID controllers in maintaining the stability of a multicopter drone. The results showed that by altering the gain values based on the different conditions of the disturbances, one can achieve a stabile drone. During further testing, it was observed that by adjusting PID gain values, the stability of the drone can be achieved within a specific fixed measure of time for a changing number of disturbances, even in tough conditions, which include wind speed and change in direction.

Madokoro et al. [60] illustrate in a comprehensive study a drone with advanced mobility on which four prototype brackets were developed. These prototypes include optimized sensors, devices and a camera, which work together as an integrated system platform. The sensors and communication system were employed as a new platform for atmospheric measurements at in situ locations, including the development of a wireless communication system for long distances and also a system for monitoring and visualizing in real-time the in situ local area measurements. The study was focused on gathering data regarding atmospheric phenomena and related environmental information, especially particulate matter (PM), as a major cause of air pollution. The obtained results were satisfactory, though, as a forthcoming design, based on regular flight measurements, and it is necessary to validate the resilience of the suggested system and its stability for long-term operation. 

The novelty of the present study, from the sensors point of view, is the relatively economical and largely accessible equipment of the hexacopter, in two versions, in terms of avionics equipment, respectively photo/video acquisition components and telemetry data transmission and reception.

### 2.3. Experimental Procedures

Megayanti et al. [61] describe the mathematical modelling and implementation of a command-and-control system for a hexacopter employed to monitor radioactive–chemical–nuclear contamination using fuzzy logic. For accurate tracking of a trajectory, the hexacopter requires a high-performance altitude and attitude controller for its in-flight movement, since in conditions of external disturbances it introduces wind. In the first step, the dynamic equation of the hexacopter is developed using Newton–Euler equations, and in the second step, a solution consisting of a PID controller combined with fuzzy logic is proposed in order to include correction signals to eliminate positioning errors of the hexacopter when moving. Before implementation on the drone, the effectiveness of the proposed method was verified using a software-in-the-loop (SITL) robotic operating system (ROS) simulation environment together with the Matlab matrix calculation utility. Based on the numerical simulation and experimental results and using the fuzzy–PID intervention algorithm, the following parameters were improved: faster transient response hexacopter trajectory tracking performance, smaller errors in maintaining the steady state of the system, faster settling times to transient changes and better and more robust static and dynamic performance under disturbances introduced by different wind speeds. 

Sharipov et al. [62] implemented a mathematical model of a hexacopter control system. Employing the Matlab/Simulink environment, it was possible to mathematically simulate the dynamics of the forces acting on the hexacopter rotors by inserting external disturbances: wind forces alongside one of the hexacopter axis. The block for estimating the model parameters is programmable and performs the computations using the theoretical formulae developed previously. Other aspects also treated in the paper were the problems associated with the selection of the optimal control for the hexacopter when flying along the path in the occurrence of wind. The attitude of the hexacopter in flight is adjusted using PID controllers because stabilization must be provided on all the axes of the drone during flight. Thus, four PID controllers must be implemented: one controller for roll motion stabilization, the second controller for pitch motion stabilization, the third controller for yaw motion stabilization and the fourth controller for hexacopter altitude stabilization. The stabilization of the hexacopter at a certain altitude was considered by Toledo et al. [63]. The Ziegler–Nichols method is employed to adjust the parameters of the PID controllers. 

Wen Fu-Hsuan et al. [64] present an analysis and management strategy for hexacopters during fixed-point hovering manoeuvres in the event of one or more engine failures. The study suggests keeping the deviation between input and output values unchanged by reallocating the thrust forces to the rotors. Simulations are performed on a hexacopter in different fixed-point flight modes [65]. Linear dynamics problems of the hexacopter are analysed and subsequently numerically validated for the unique nonlinear dynamics. If failure of one of the hexacopter motors occurs, the study proposes an allocation matrix to reallocate the lift forces to the functional motors. The study takes into account seven cases of engine failure; the conclusions derived from analytical analysis show that reduced control for emergency landing is achievable in four scenarios at the linear level, and for the other three scenarios, the drone is completely uncontrollable. To demonstrate the validity of the recommended algorithm, the paper also presents numerical simulations.

Derawi et al. [66,67], Poksawat et al. [68] and Zheng et al. [69] present the mathematical modelling, estimation, attitude (drone position) control and altitude control of a hexacopter. Their works present the following contributions: First, mathematical modelling is performed, based on which the equations of the hexacopter model are obtained. Again, the problem of rigid solid dynamics is pointed out by the study being performed on a hexacopter with an “X” configuration. The modelling is carried out employing the homogeneous transformation matrix, the Euler angles (ϕ—roll, θ—pitch, ψ—yaw) and the two reference systems, one associated to the drone frame and one inertial, grounded. It also introduces certain notions about the aerodynamic forces and moments that act on the drone frame. The dynamic model of the hexacopter illustrates the translational and rotational motions in response to the thrust generated by each rotor. A new approach for real-time drone attitude estimation is proposed by Benzemrane et al. [70] and Benzerrouk et al. [71] using a complementary nonlinear observer based on a special orthogonal group of rotation matrix SO (3)—special orthogonal—compared to the conventional extended Kalman filter (EKF). The works propose an attitude controller based on a PI and PID inner–outer loop structure, aiming to lead to faster response and improved strength at transitory response, while the altitude controller proposed in the paper is based on a standard closed-loop PID control system. The hexacopter employed in the tests is equipped with low-cost sensors (an inertial measurement unit (IMU) sensor modelled by Neumann and Bartholmai [72], and Sushchenko and Beliavtsev [73] and a barometric pressure sensor). Finally, through the experiments performed (flight manoeuvres inside a building and flight manoeuvres performed in outdoor free space, also taking into account the effects of disturbing factors, in particular, wind), Heise et al. [74], Dong et al. [75] and Lee et al. [76] demonstrate the efficacy of the suggested attitude of the observer, and Seah et al. [77] determine the attitude controller and altitude controller in real flight conditions, both in indoor and outdoor environment. 

All the analysed papers present theoretical and laboratory-performed tests. In order to demonstrate the full functionality of the proposed hexacopter, the present work addresses a compound laboratory and in situ test procedure, comprising both ground and flight tests in a new perspective. 

### 2.4. Multirotor Drones Stability Assessment Based on FEM Approach 

When discussing the hexacopter stability [78,79,80,81,82], aspects related to the structural components of a hexacopter platform have to be considered. In the case of FEM analysis, this frequently focuses on structural stiffness and stability and requires a detailed assessment of the hexacopter model in a synergic connection between the virtual CAD model and FEM codes. The results matter not only in the decision-making process regarding the design of the hexacopter structure but also to the flight stability and the position control on the trajectory.

Reducing the drag force remains one of the main challenges in UAV aerodynamics research, as battery consumption can be significantly reduced if the drag decreases. Felismina et al. [83] analyse the aerodynamic behaviour of a quadcopter equipped with a seeding device in order to determine the appropriate bank angles (0°, 15° and 30°) for take-off and drone flight evolution during the seeding operation. Moreover, the work aims to define a suitable flight plan to increase the battery range. Aerodynamic results demonstrate that for take-off, the 30° tilt represents the most favourable aerodynamic position, due to the lower drag force that occurs during climb. In terms of the drone’s behaviour during seeding, the 0° tilt is the one that creates a lower frontal drag and a lower drag force coefficient, respectively.

Lei et al. [84] discuss the aerodynamic performance of a hexacopter with different rotor spacings. The hovering flight efficiency of the drone is analysed by performing experimental tests and numerical simulations. A number of indices characterising the aerodynamic performance of the hexacopter are analysed theoretically, followed by tests and simulations on a hexacopter drone with different rotor-spacing ratios in relation to propeller size (i = 0.50, 0.56, 0.63, 0.71, 0.83). Using a custom-conceived test platform, the thrust, power load and hover flight efficiency of the hexacopter were obtained. Finally, CFD simulations were performed to obtain the fluid flow, pressure and velocity contour distributions of the hexacopter. The results show that the aerodynamic performance of the hexacopter drone varies by changing the rotor spacing. It was also observed that the thrust force increased by 5.61% and the overall efficiency increased by about 8.37% at i = 0.63 for the working mode (2200 RPM), indicating that the rotor spacing ratio at i = 0.63 achieved the best aerodynamic performance. 

The design and development of a hexacopter capable of lifting a high payload has been investigated by Suprapto et al. [42] to ensure a stable attitude in flight. The evaluation focusses on the frame displacement and stress analysis to ensure the expected payload. In [85], experimental and CFD simulation tests for a small size UAV model to test wind influences at low speeds were reported. The study is comprehensive, but the prototype geometry as well as the flow regimes were found to be below the common level of UAV applications. 

The flow regimes of air streams from the upper to the lower surface at different fixed-point flight altitudes were simulated and analysed by Zheng et al. [86] for plant protection applications. Although the study is substantial, the results are particular to the field of agricultural engineering. 

In conclusion, many FEM simulation attempts have been reported on hexacopters, but the most interesting results for this type of platform have been achieved by software developers to demonstrate the capabilities of the solvers, as simulation in this case is still considered a challenge. In the case of the six rotors, the rotational domains of the propellers are so close to each other that the narrow space induces even more modelling and computational difficulties. From this point of view, the current research aims to bring a new perspective to the scientific literature.

## 3. Hexacopter Platform Architecture

**Figure 1:**
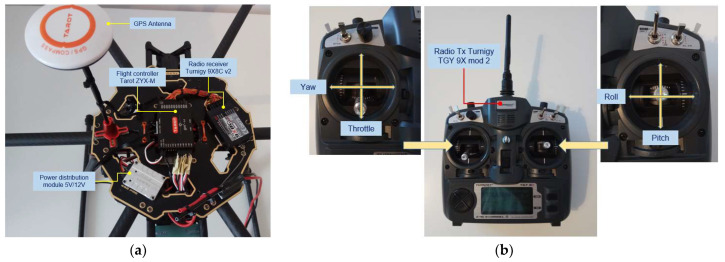
Variant v1 of the hexacopter. (**a**) Equipment; (**b**) Radio control.

The proposed hexacopter is presented in two equipment variants (v1 and v2), with two different sets of avionics equipment. Variant 1 (v1) (Figure 1) illustrates the Tarot ZYX-M avionics kit composed of the following: Tarot ZYX-M flight controller (AP—autopilot), 5V/12V voltage distribution module, GPS antenna sensor, status LED and radio receiver Turnigy 9X 8C v2 sensor on eight channels, frequency 2.4 GHz. A Turnigy Multistar 4-cell LiPo battery in 4S1P configuration with a capacity of 6600 mAh was employed as the drone power source. The radio control is model Turnigy TGY 9X, mode 2 with nine transmission channels, which is paired with the Turnigy 9X 8C v2 radio receiver mounted on the drone. For this variant, no data transmission–reception equipment was endowed for telemetry and video signals from drone to the operator. This variant is employed only for the preliminary testing of the normal operational parameters, on the ground and during the flight of the hexacopter, without a detailed analysis of the flight outputs.

**Figure 2:**
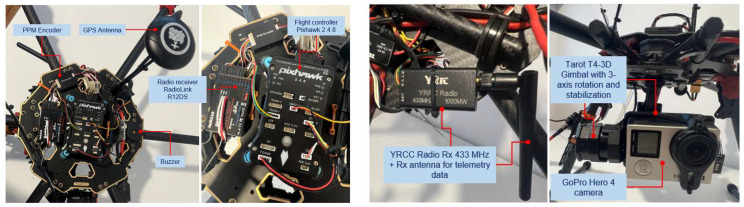
Variant v2 of the hexacopter.

Figure 2 depicts the second version of the hexacopter, composed of the flight controller AP Pixhawk 2.4.8 sensor, a PPM protocol encoder sensor that allows the encoding of eight signals using the pulse width modulation (PWM) protocol in a single signal employing the pulse position modulation (PPM) procedure; loudspeaker, for AP status beeps; fail-safe on/off switch for protection against accidental starting of the motors; data transmitter telemetry transmitter YRRC at the 433 MHz frequency and 1000 mW power for the transmission of telemetry data on the ground, paired with the signals telemetry on the ground receiver, model YRCC, which transmits the video signal on 32 channels at a 5.8 GHz frequency; 600 mW power, for video signal transmission from the GoPro Hero 4 model camera mounted on the three-axis rotation gimbal, Tarot T4-3D model; a 12-channel RadioLink R12DS radio receiver; 2.4 GHz frequency for radio command reception from the transmitter built into the control box at the ground operator; GPS signal reception antenna; and the ReadytoSky model. 

Both variants were mounted on the same hexacopter frame structure, made of carbon fibre and consisting of two central plates of the frame between which six supported arms are fixed. At the arm ends, the motors and electronic speed controllers are mounted on six special supports. The landing gear is composed of two tubular structures mounted in the form of the letter T at a specific angle to the end plate of the frame. At the bottom of the lower plate, a bracket is mounted for fixing the battery. In the v1 variant, an additional support is mounted on the right arm of the landing gear for fixing the video transmitter. The carbon fibre provides the drone frame with elasticity, i.e., increased resistance to deformations, stresses, bending and a reduced structural mass of the platform. However, a drawback arises from the fact that carbon fibre attenuates the strength of the transmitted/received radio signal. That is why it is necessary to carefully choose the location of the radio/video signal transmission–reception equipment on the hexacopter or its proximity by mounting spacers.

**Figure 3:**
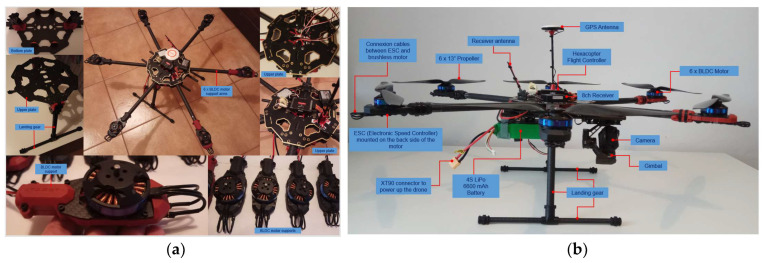
Hexacopter variant v1. (**a**) Components; (**b**) Assembly.

**Figure 4:**
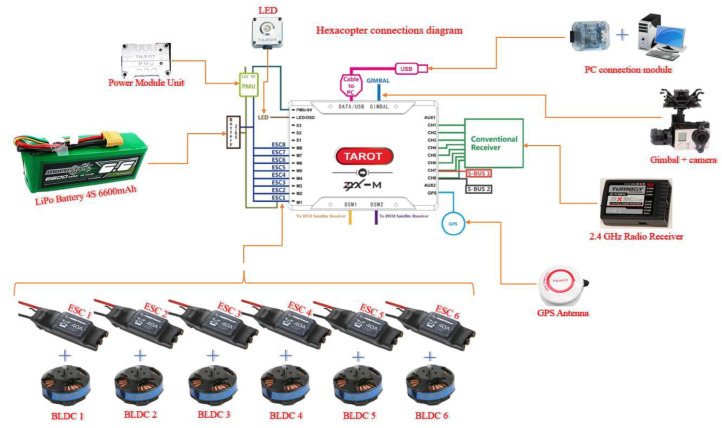
Connections diagram for hexacopter variant v1.

**Figure 5:**
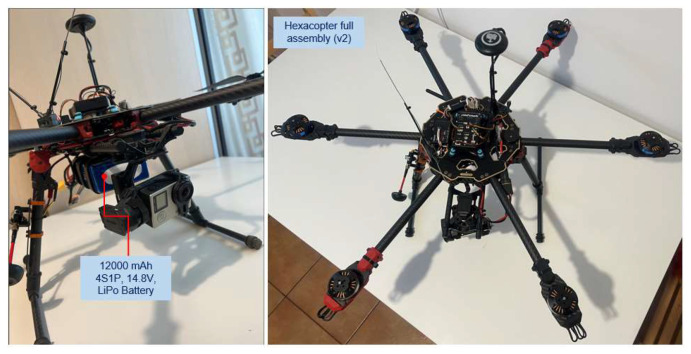
Hexacopter assembly for variant v2.

Figure 3, Figure 4 and Figure 5 depict the described design details both focused on components and on the entire equipment assembly for variants v1 and v2 (with 4S1P LiPo battery, 14.8 v, 12,000 mAh). 

**Figure 6:**
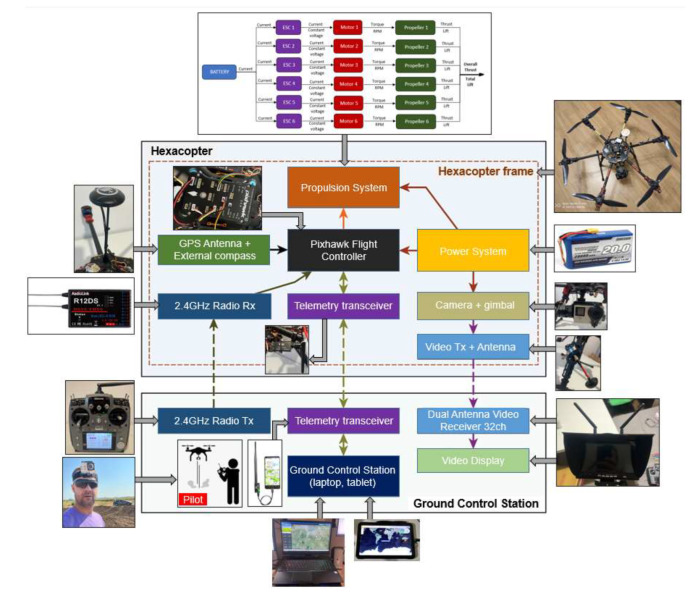
Blocd diagram of hexacopter platform.

The block diagram of the hexacopter platform architecture is presented in Figure 6 illustrating the main components of the hexacopter, respectively the command-and-control ground station and the relationships between them. Hexacopter telemetry data are transmitted via a YRCC transmitter equipped with an antenna operating at 433 MHz. On the ground, an YRCC receiver is equipped with an antenna paired with the one placed on the hexacopter and at the same operating frequency. The receiver can be connected to a mobile device (tablet or smartphone) or a laptop, on which a GCS (ground control station) platform is installed. 

**Figure 7:**
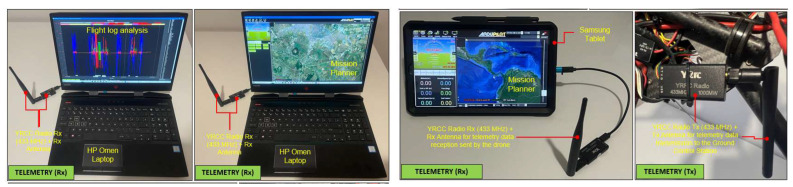
Arrangement of the telemetry kit on the drone and on the ground.

The two components of the telemetry kit are illustrated in Figure 7: the transmitter mounted on the drone and the receiver in two connection options (Samsung tablet and HP Omen laptop), on which the Mission Planner GCS was installed. 

**Figure 8:**
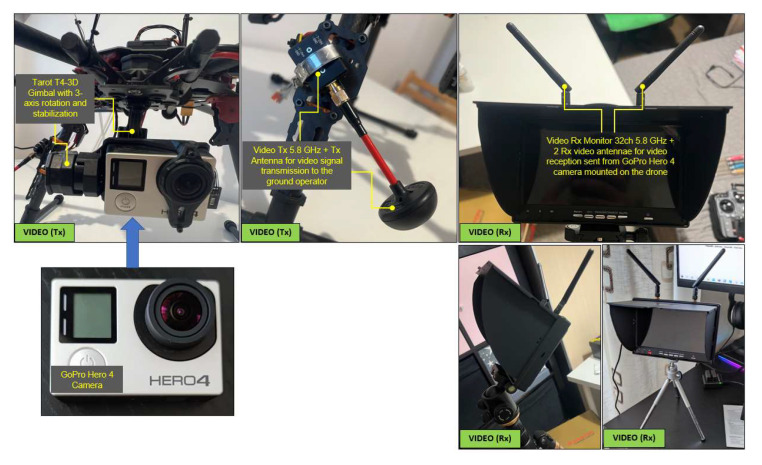
Video signal transmission–reception chain from hexacopter to the operator.

The video signal from the hexacopter is either stored on the GoPro camera’s internal microSD card (when it has to operate in record mode) or transmitted in real time to the ground by means of the following chain: the GoPro camera is connected to the Tarot T4-3D gimbal via a special dedicated connector; the video signal is then transmitted to a 32-channel antenna operating in the 5645–5945 MHz frequency range. This communicates with a dual receiver (two built-in antennas for better signal reception) on 32 channels, on the same frequency of 5.8 GHz, and the image is displayed on a 7″ HD monitor. Following laboratory tests, for optimal operation of the transceiver chain, the transmitter was set to channel 4 (5645 MHz), and the receiver was set to channel 5 (5885 MHz), according to the frequency matrices in the specifications of each component. Figure 8 illustrates the composition and location of the video transceiver system from the hexacopter to the operator. The HD video monitor with the built-in receiver is shown in the tripod-mounted version, but it can also be mounted on the operator’s radio remote control for easy observation of real-time images and gimbal control to obtain the desired frame during the surveillance, reconnaissance, investigation and shooting missions.

**Figure 9:**
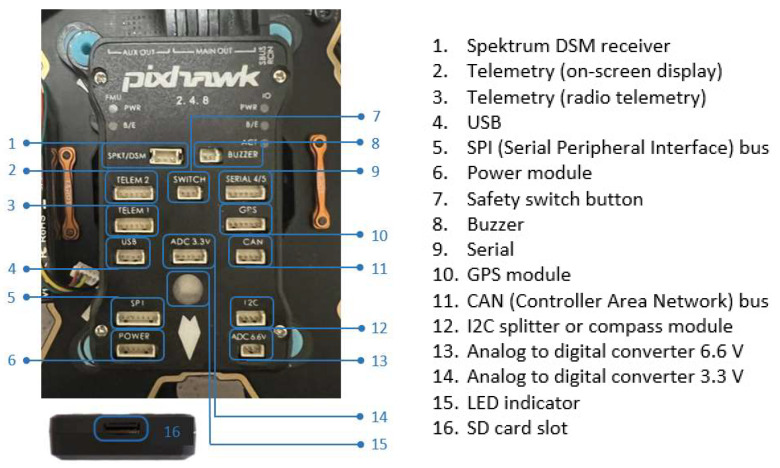
Pixhawk 2.4.8 flight controller and the peripheral connection interfaces.

The Pixhawk 2.4.8 flight controller (FMUv2) installed on the hexacopter v2 version, with the interfaces to various peripheral equipment, are illustrated in Figure 9. 

The flight controller hardware components are the following:

- System-on-Chip STMicroelectronics STM32F427 Cortex-M4F 32-bit main microcontroller, operating frequency 180 MHz, RAM: 256 KB SRAM (L1), 2 MB Flash memory for writing instructions.
- System-on-Chip STMicroelectronics STM32F100 Cortex-M3 32-bit, 24 MHz operating frequency, 8 KB SRAM (L1), 64 KB Flash memory for writing instructions.
- Embedded sensors on the motherboard: ● a 3-axis STMicroelectronics L3GD20H 16-bit gyroscope sensor; ● a 14-bit STMicroelectronics LSM303D accelerometer/magnetometer sensor; ● an Invensense MPU-6000 3-axis accelerometer/gyroscope sensor; ● a TE Connectivity MEAS MS5611 barometer sensor.

Also connected to the flight controller is an external GPS antenna/bus module consisting of a Ublox M8N GPS receiver sensor and a Honeywell HMC5883L digital compass sensor.

For the subsequent analysis of the flight parameters both on the ground and in flight an ArduCopter firmware, version v4.x, was employed and installed on the Pixhawk 2.4.8 AP motherboard. A laptop and a tablet were utilized for the ground control station on which the mission planner platform was installed and employed. In the v2 version, the drone powering was achieved with three Turnigy batteries, LiPo type with four cells, in 4S1P and 4S2P configurations, maximum supported current 12-24C, with capacities 12,000 mAh, 16,000 mAh and 20,000 mAh. 

The hexacopter presented in the two variants, equipped with avionics components and sensors, can be employed in a wide range of civil applications, as well as in the field of homeland and national security. The solutions developed represent a relatively economic option in terms of the component acquisition costs and integration. 

## 4. Laboratory and In Situ Measurement Results and Discussion

Tests were carried out, both those on specialized online platforms specifically tailored for the multirotor drone segment as well as extended laboratory, ground and flight tests to confirm the optimal real-world operation of the hexacopter, in accordance with the original design; practical realization of the hexacopter; and choice and integration of avionics components, command and control, video acquisition and telemetry data, taking into account a future development of a variant of equipment for command and control of the hexacopter out of direct line of sight (BVLOS).

### 4.1. Laboratory Tests

To ensure that all the components were correctly chosen and the hexacopter will perform as expected, preliminary laboratory tests were conducted using dedicated built test stands and testing equipment. 

**Figure 10:**
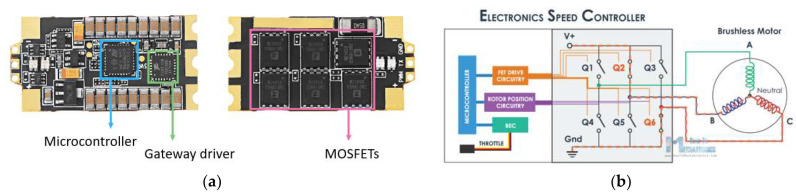
The ESC architecture and simplified diagram of ESC operation. (**a**) ESC general architecture; (**b**) Simplified diagram of ESC operation.

Aside from the mentioned sensors, the electronic speed controller (ESC) sensor must be considered due to its major importance when discussing motors functionality. The basic function of an ESC is to control the motor speed based on the PWM (pulse width modulation) signal that the AP sends to the motor, which is too weak to drive the brushless DC motor directly (Figure 10). This is achieved by the pilot operating the speed stick in the range 0–100%, and the ESC will send the power commanded by the pilot to the motor. In addition, some ESCs also perform other functions: dynamic braking, battery short-circuit protection, motor start protection and power supply (battery disposal circuit) for the radio receiver or servo motors. Unlike a general ESC, the ESC for brushless motors can act as an inverter, converting the direct current received from the battery into three-phase alternating current (AC), which is then applied to the motor. The ESC also determines the direction of rotation of the motor.

The simplified diagram of ESC, depicted in Figure 10b, employs real-time operation. The main features and parameters of the ESC mounted on the hexacopter are as follows: it is equipped with specially optimized firmware for disc-type motors and a special core program for rapid throttle response, and the refresh rate of the throttle signal supported is up to 621 Hz, making the ESC perfectly compatible with various flight controllers (if the refresh rate is higher than 500 Hz, then the ESC control signal is the nonstandard throttle signal); it is equipped with driving efficiency technology (DEO), which effectively reduces the ESC operating temperature by about 20%, improves the flight time and brings a better throttle linearity and good stability—thus the operating efficiency improves by maximum 10%. In addition, its MOSFETs have extra-low resistance, offering high performance and great current endurance, with a continuous output current of 40 A and a burst of 60 A up to 10 s.

**Figure 11:**
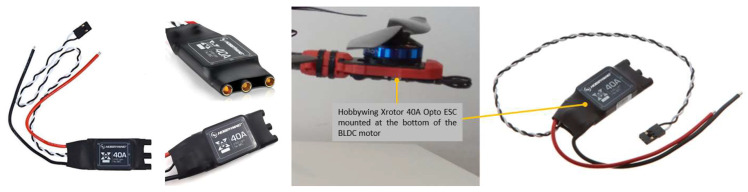
Hobbywing XRotor 40A Opto ESC.

The most important components of the ESC, as presented in Figure 11, are the microcontroller, the driver for the gateway between the autopilot (AP) and the MOSFETs and the MOSFETs, respectively. As illustrated previously, the hexacopter is equipped with six Hobbywing XRotor 40A Opto ESCs sensors, each of these being connected to each of the six BLDC motors. 

**Figure 12:**
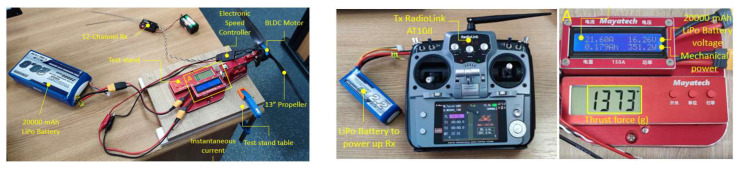
Propulsion system efficiency test stand configurations.

To determine the thrust force, and therefore the efficiency of the propulsion system, laboratory tests were carried out using the Mayatech MT10PRO 10KG test stand. A Turnigy LiPo battery, 20,000 mAh capacity, 4 cells in 4S1P configuration, voltage 14.8 V, was employed to power the ESC–motor–propeller assembly. The Tx–Rx chain was provided by a 2.4 GHz radio remote control, model RadioLink AT10II, and a 12-channel receiver, model RadioLink R12DS. The test configuration is shown in Figure 12.

**Figure 13:**
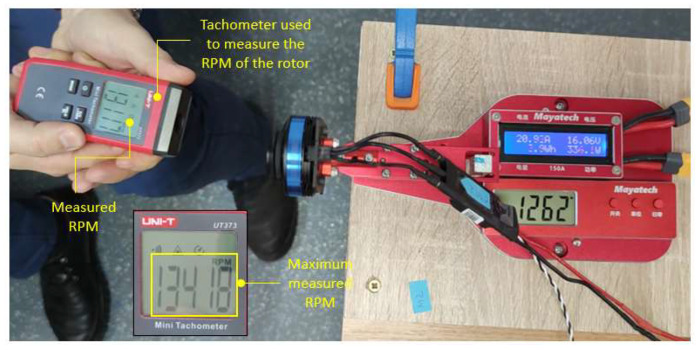
Measurement of the rotor assembly maximum RPM.

Using a tachometer, in the same test configuration, the maximum speed of the rotor assembly was determined, obtaining a maximum value of 13418 RPM (Figure 13).

**Figure 14:**
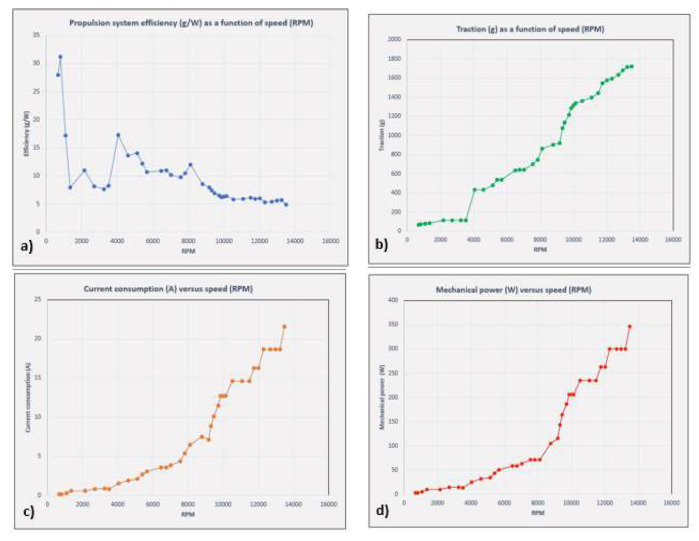
Propulsion system tests results. (**a**) Propulsion system efficiency; (**b**) Traction force as a function of RPM; (**c**) Current consumption based on RPM; (**d**) Mechanical power versus RPM.

The thrust force, current consumption, battery voltage and mechanical power were measured using the test stand (Figure 12), and the rotational speed using the tachometer (Figure 13). The results are plot in the graphs presented in Figure 14. 

Therefore, the results obtained were as follows:

- The maximum thrust force produced by the rotor assembly, measured on the stand, was approximately 1.718 Kgf ≈ 16.84 N.
- Maximum speed measured by the tachometer—13418 rpm.
- The efficiency of the propulsion system decreases with increasing rpm. In the idling zone, at 30–40% rpm, the efficiency reaches a value of 13–14 g/W (≥6 g/W—high-efficiency drone). In the 50–75% rpm range, which is equivalent to operating the drone in hover and light horizontal manoeuvres, the efficiency decreases to a value of 6.49 g/W (≥6 g/W—high-efficiency drone). In the speed range of 85–100%, the efficiency further decreases to a minimum value of 4.96 g/W (4 ÷ 6 g/W—low-efficiency drone).
- With increasing speed, the current consumption increases proportionally, reaching a measured current value at 100% speed of 21.6 Ah.
- The mechanical power produced also increases to a value of 346.2 W at 100% speed.

**Figure 15:**
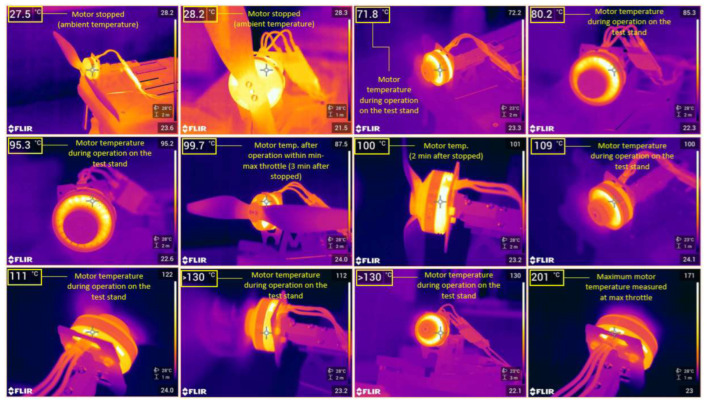
Measurement of motor temperature during operation on the test stand, within 5–100% throttle range.

The optimum operating temperature of the BLDC motors is of critical importance as high temperatures can lead to premature engine damage and failure. Thus, it is necessary to test the operating temperatures at idle, midthrottle and maximum throttle to determine the RPM ranges where their performances are at best. During the tests on the test stand, motors temperatures at different RPM ranges were measured employing a FLIR E86 thermal imaging camera (Figure 15). 

By analysing the results, the following points of interest were found:

- At idle, with the throttle stick at 30%, for a 3–5-min interval, the motor temperature reached 40 °C.
- At idle, with the throttle stick at 50% for 3–5 min, the motor temperature reached 60 °C.
- In maximum mode, with the throttle stick at 100%, for 3–5 min, the temperature reached over 200 °C, which means that it is only desirable to operate the drone in maximum mode for very short periods, around 10–15 s, to avoid these temperature increases in the motor windings, which can eventually lead to burn-out and thus their permanent damage.

### 4.2. Online Testing Platforms Results

In order to find the best configuration to ensure a stable flight with the best range within safe flight conditions, several variants of equipment, including sensors, propellers and batteries, were tested using online platforms specialized in the multirotor drone segment. This section presents the best results for the proposed hexacopter in terms of stability and flight range employing the *xcopterCalc* simulation platform, one of the most popular tools in terms of configuration simulations for multirotor drones. 

**Figure 16:**
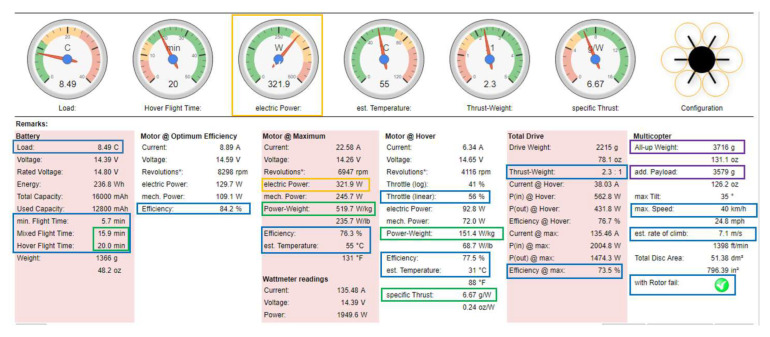
Results obtained after running the simulation using *xcoperCalc* platform.

Similar results for the hexacopter were obtained for both variants, were two different flight controllers and avionics components were employed, Tarot ZYX-M and Pixhawk 1, respectively, as presented above. Figure 16 illustrates the results obtained in variant 1 of the hexacopter, where Tarot ZYX-M flight controller and subsequent avionics components were utilized. The configuration used was as follows:

- Frame size is 695 mm and is made of carbon fibre epoxy resin with a total mass of only 833 g, while providing increased shock and vibration resistance.
- The propellers were 13’’ with 5.5″ pitch—the size of the drone frame limits the mounting of propellers with a maximum diameter of 13″.
- Four-cell LiPo battery capacity—16 Ah, in 4S2P configuration with 12-24C C-rating, 14.8 V nominal voltage.
- Flight testing of the HDT was simulated at an altitude of about 85 m above sea level (Bucharest altitude), at a temperature of 22 °C and at an atmospheric pressure of 1010 hPa (757.5 mmHg).
- Electronic speed controllers (ESC) can withstand a maximum current of 40A and have an internal resistance of approximately 0.0006 Ohm and a mass of 26 g each.
- The hexacopter has a three-axis rotating and stabilizing gimbal; it has a mass of 178 g and consumes approximately 0.05 A.
- Tarot 4006/620KV BLDC motors produce 620 rpm/V and have an internal resistance of 0.126 Ohm and a mass of 82 g each.

After running the simulation, the following results were obtained, as illustrated in Figure 16.

The following conclusions were drawn:

- Load on the battery (load) is 8.49C (which means a continuous load below 12C A of the battery, i.e., 8.49 × 16A ≈ 136A < 12 × 16 ≈ 192A).
- A considerable increase in flight time to 15.1 min for combined flight and 20 min for hover flight, compared to lower capacity batteries used in previous tests.
- For optimum motor performance, a slight increase in efficiency from 84.1% to 84.2% is obtained; for fixed-point flight, a speed of 4116 rpm is obtained. The motor speed increases from 48% to 56% of capacity (which is a good result), a power-to-mass ratio of 151.4 W/kg, an efficiency of 77.5% and a temperature of only 31 °C. However, as an element to be taken into account, an increase in power (at engine input) to 321.9 W (but only at maximum engine speed) is noted.
- The thrust-to-mass ratio in this case is 2.3:1 (>1.8—very good value).
- The specific thrust of the propellers is 6.67 g/W, so high efficiency.
- Additional equipment with a mass of about 3.6 kg can be attached.

**Figure 17:**
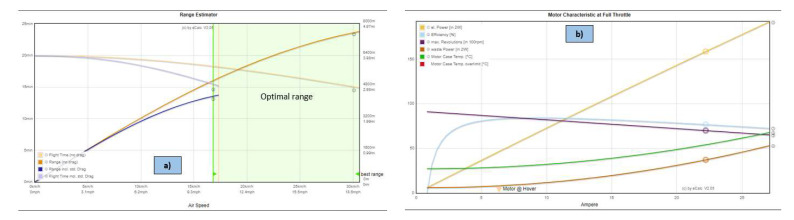
Online tests. (**a**) Range estimator; (**b**) Motor characteristics at full throttle.

Figure 17 depicts two graphs (a) and (b), obtained after running the simulations, showing data regarding flight distance, speed and engine characteristics at maximum speed.

The following conclusions were summarized:

- The maximum speed is 40 km/h, and the ascent rate of 7.1 m/s;
- The maximum flight time (without drag) is about 20 min;
- Maximum flight time (with drag) decreases to 15.1 min;
- The maximum flight distance (without drag) is approximately 7600 m;
- The maximum flight distance (with drag) is approximately 4400 m;
- The best performance for the hexacopter is achieved within the speed range 17 ÷ 31 km/h;
- From Figure 16, it can be observed that the engines succeeded to operate in all speed ranges at an acceptable temperature of maximum 55 °C, which is very good for flight stability and proper functioning of the avionics and airborne sensors.

### 4.3. In situ Ground and In-Flight Experiments

The results presented in this section were attained from tests carried out for the hexacopter v2 configuration, equipped with the Turnigy 12000 mAh 12-24C LiPo 4S1P battery. 

#### 4.3.1. Hover Flight

The purpose of these tests is to ensure, verify and prove the appropriate functioning of the hexacopter in the proposed configuration, both in terms of the structural design and the avionics components, especially the employed flight controller. These tests are divided into two main categories: ground and flight tests. The purpose of the ground test is to ensure that the drone’s structure and avionics systems comply with the requirements so that the hexacopter will perform the flight as expected.

##### Ground Test

This test consists of:

- Inspection of the structural integrity of the drone. Each joint of the structural elements is checked and must be well secured to ensure its rigidity.
- Checks of the weight and the drone equilibrium. These checks provide information on the location of the actual centre of gravity in respect to all three axes X, Y and Z. The centre of gravity location affects the performance and stability of the drone in flight.
- Examination of the avionic systems operation: controller, navigation, power supply, video system, telemetry data transmission system and wiring. All data concerning the operating limits of the equipment must be memorized in order to avoid undesirable events such as maximum drone range, maximum operating range of the radio controls, battery capacity, power consumption of the various electronic components, maximum authorized flight altitude and legislative aspects concerning the operation of the drone in certain areas, depending on the geographical layout. In the case of autonomous flight following a preprogrammed route, the flight controller has programmed the flight scenario, the flight parameters and the failsafe measures required in the event of emergencies such as the loss of radio link between the drone and the operator, battery voltage falling close to the critical value and a motor shutdown.
- Test of the motor’s operation by simple on/off commands to ensure the rated static performance based on throttle stick position, increasing the speed incrementally up to 10–15% and checking their operation, oscillations, noises, proper propeller rotation directions.
- Telemetry data link tests between the drone and the mission planner ground control station. This ensures the stability of the radio link between the drone and the operator. With the help of the control station, the operator can either plan autonomous flights on preprogrammed routes or intervene in the control of the hexacopter in emergency conditions if the radio control is not used.
- Weather condition checks: wind speed, temperature, precipitation and atmospheric pressure. This is an extremely important step in planning a flight, as there are limitations to operating the hexacopter.

**Figure 18:**
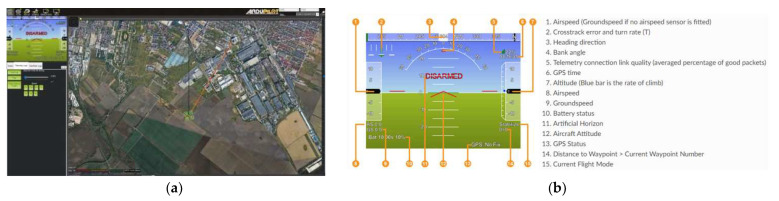
Mission planner ground control station. (**a**) Main window; (**b**) HUD window.

For the GCS, the mission planner platform was employed, whose main interface is illustrated in Figure 18a, which also shows the map of the test location. Figure 18b depicts the HUD window, which provides valuable data for the operator during flight stage and also during ground operations.

**Figure 19:**
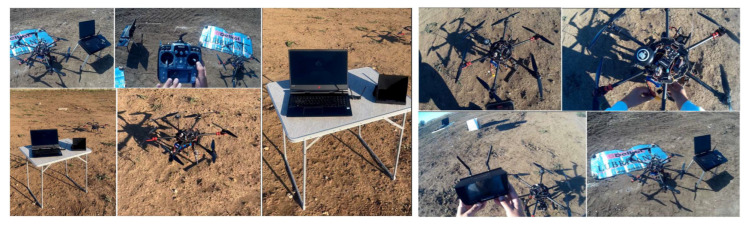
Employed open area test rigs.

**Figure 20:**
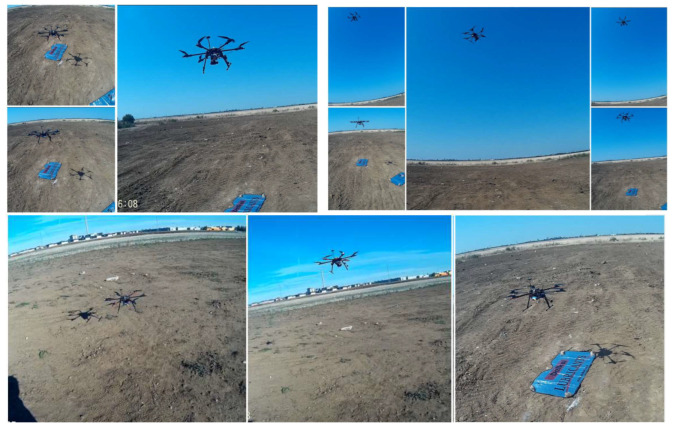
Hexacopter in stationary flight at a fixed point—flight stages.

Figure 19 pictures the test stands, the ground control station and the test location, while Figure 20 encompasses details during the execution of the hexacopter manoeuvres: take-off, climb, hover, descent and landing.

**Figure 21:**
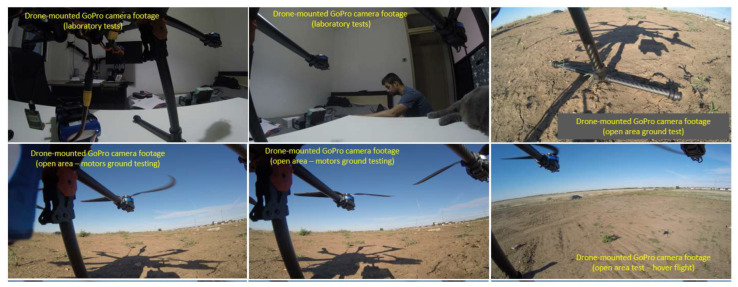
Drone-mounted GoPro camera footage, on the ground and in flight.

**Figure 22:**
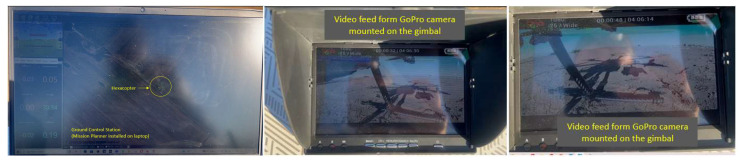
Mission planner interface. Images acquired by GoPro camera mounted on the hexacopter.

Images of the drone on the ground and in flight received from the GoPro Hero 4 camera mounted on the hexacopter are illustrated in Figure 21, while Figure 22 represents the mission planner interface with the layout of the hexacopter on the test location map and the video received from the GoPro camera on the built-in dual receiver monitor.

##### Hover Flight Tests

**Figure 23:**
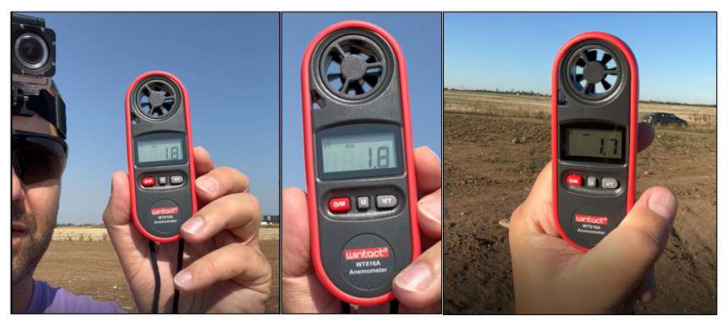
Wind speed measurement with anemometer.

After the completion of the ground experiment, the hexacopter was tested in stationary flight. The tests were carried out in a plain area without obstacles around, within a radius of 5 km, in order to avoid the occurrence of unpleasant events such as drone crash, property destruction or person injuries. Regarding the wind speed at the test site, days with low wind speed of 1–2 m/s were chosen, measured with an anemometer (Figure 23).

In this manoeuvre, after the operator has given the command to increase the engine speed, the speed stick is kept in the 50–75% rpm range (for the hexacopter configuration), and the lift force required to lift the drone off the ground and keep it stationary is obtained. The lift force is created by the rotation of the six rotors, which revolve at the same rpm during the vertical climb, while the hexacopter tries to stabilize its attitude. During the vertical lift, until the altitude set by the operator is reached and in respect to the command given by the operator, the hexacopter attitude PIDs apply corrections to maintain the position of the drone within the values of the commanded parameters. When stabilizing the hexacopter at a given altitude, the operator keeps the speed stick in the appropriate speed range, and the PID altitude controller applies the necessary corrections to the thrust of the engines to maintain the drone at the desired altitude. 

**Figure 24:**
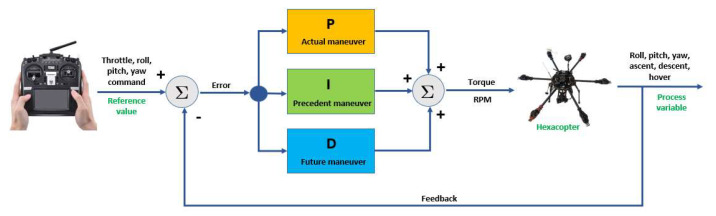
Closed-loop PID scheme—general approach.

The general architecture of the closed-loop PID controller is depicted in Figure 24.

In the case of the physically designed hexacopter equipped with a three-axis gimbal and a photo/video camera, the drone must be able to maintain its position at a fixed point in order to carry out surveillance, reconnaissance and photography missions. For this purpose, when using the manual radio control by the ground operator, it is recommended to operate it in the following modes: stabilize, in which the PID controllers automatically adjust pitch and roll; alt hold, in which the PID controllers automatically adjust pitch and roll and maintain drone altitude; and RTL (return to land) for emergency cases. Loiter can also be employed (semiautonomous flight—the PID controllers automatically adjust the drone’s altitude and position; the hexacopter uses GPS for movement), PosHold (similar to loiter mode, but when the roll and pitch sticks are not centred, the operator controls the two movements) and land (the hexacopter descends and lands directly, without returning to the take-off point). Auto mode is utilized for autonomous movement along a predefined flight path. For PID controller tuning procedures, AUTOTUNE mode is employed after sensor calibration procedures (accelerometers, gyroscopes, magnetometers) and initial tuning.

In the case of fixed-point tests, take-off was performed in stabilize mode, which was then switched to alt hold mode and subsequently to RTL mode.

#### 4.3.2. Hexacopter Flight Parameters Extracted from Sensors during Hover Flight

**Figure 25:**
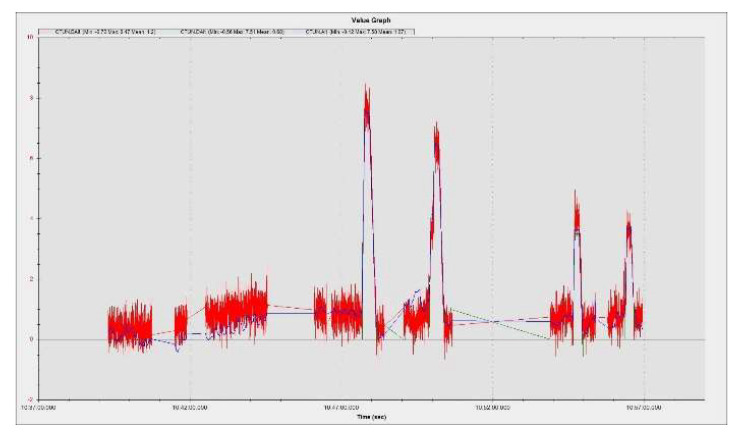
Hexacopter programmed and recorded altitude.

The drone was raised up to an altitude of 8.5 m. The EKF subsystem is responsible for generating attitude, velocity, position and altitude estimates for the drone so that the navigation and control systems can operate correctly. EKF takes the inputs from IMU, GPS and BARO sensors and integrates them to provide these assessments, one of which is the estimated altitude. This is then passed to the vehicle’s altitude control system, which attempts to align to the target altitude in altitude-controlled flight modes. Figure 25 plots the altitude reached by the drone in blue, the commanded altitude in red and the altitude measured by the barometer in green. It can be noticed that there are no significant differences between the three values. The maximum recorded difference between programmed and measured values is less than 1 m, representing less than 10% of the real value.

**Figure 26:**
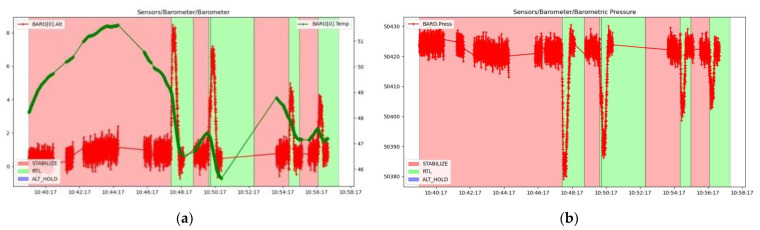
Hexacopter measured parameters. (**a**) Altitude and ambient temperature; (**b)** Ambient atmospheric pressure.

The graph extracted from the MAVExplorer platform (Figure 26a) depicts the altitude at which the drone was lifted and the ambient temperature at the test site in Fahrenheit degrees. The graph presented in Figure 26b shows the in situ atmospheric pressure (Pa) extracted from the data measured by the barometer with which the drone AP is equipped.

For optimal operation of the hexacopter, radio control calibration was initially performed using the GCS mission planner. The minimum maximum PWM signal duration limits for the employed channels by the RadioLink AT10II radio control, mode 2 (motor stick is located on the left side), operate within the range 1000–2000 µs. The channels are assigned as follows: Channel 1 (CH1)—roll, Channel 2 (CH2)—pitch, Channel 3 (CH3)—throttle, Channel 4 (CH4)—yaw, Channel 5 (CH5)—flight modes (stabilize, alt hold and RTL), Channel 6 (CH6)—engine kill switch mode. 

The PWM signal is utilized to control the pulse width modulation signal for each electronic speed controller that is connected to each of the six motors. The PWM signal is a periodic square wave signal with a period of 20 ms, which means it has a refresh rate of 50 Hz. Each cycle of the PWM signal lasts for 1–2 ms high level (1000–2000 µs), which is the control value of that channel. In the case of speed, 1000–1100 µs corresponds to 0 speed of the hexacopter, and 1900–2000 µs corresponds to maximum speed.

**Figure 27:**
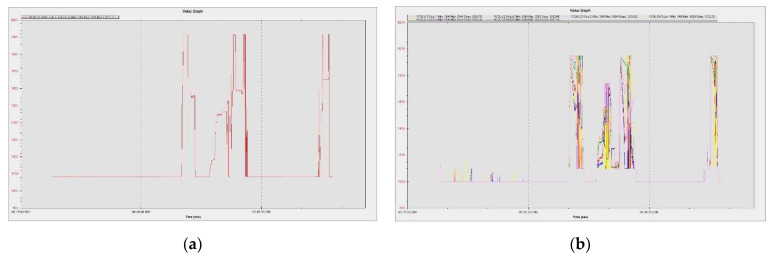
Tests performed on the motors without propellers. (**a**) Speed command given by the operator; (**b**) Engine response to the operator command.

Prior to ground and flight manoeuvres, laboratory tests were performed on the motors without propellers to verify their operation within parameters. Figure 27a illustrates the command given by the operator from the radio control, in the range 1083–1916 µs, as it was previously calibrated, and Figure 27b shows the response of the ESC-controlled motors in respect to the operator’s command to increase speed. It can be noted that the engines operate within the appropriate parameters, with values between 1000–1950 µs, and respond directly to the command given by the operator.

**Figure 28:**
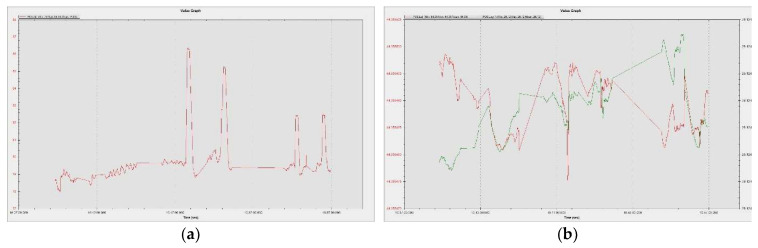
The altitude of the test site. (**a**) Operating altitude of the in situ location; (**b**) Test location.

Figure 28a depicts the altitude of the test site, which is 79–80 m above sea level (mean sea level). The peaks of the graph represent the altitudes to which the hexacopter was lifted during the manoeuvres. In Figure 28b, the geographic coordinates of the location (maximum altitude of the hexacopter stationary at the fixed point—8.5 m) are illustrated.

**Figure 29:**
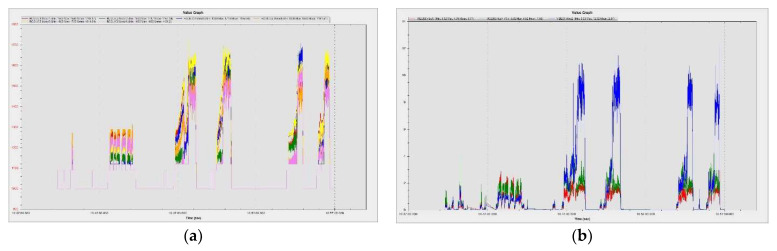
Results processed with the online platforms. (**a**) Engines response to the lift command; (**b**) Accelerometer (0) vibration recordings.

The results were processed with the mission planner and MAVExplorer platforms. Figure 29a depicts the command given by the ESCs to the drives in response to the operator moving the speed stick in the 50–75% range. The motor increases the rotational speed to a value at which the hexacopter becomes detached from the ground and begins to lift until the operator clamps the stick at a certain percentage of the speed. The operation of the motors is observed in the range 1000–1725 µs, as the rotational speed range does not reach 100%, leaving room for additional manoeuvring if needed. It is noticed that there are small delays in response times between 100 and 200 µs, representing less than 10%, possibly due to the natural frequencies of the motors or the structural elements on which they are mounted. High vibrations can cause wrong accelerometer altitude and horizontal position estimations, leading to problems in maintaining altitude. The hexacopter may start an uncontrolled lift manoeuvre without the operator being able to intervene. Position control problems in flight modes such as loiter, PosHold and auto may also occur. Vibrations are best visualized by plotting the VibeX, VibeY and VibeZ values in the VIBE menu. These represent the raw vibration values before being filtered by the accelerometers. Vibration levels below 30 m/s^2^ are normally acceptable. Levels above 30 m/s^2^ can cause problems, and levels above 60 m/s^2^ nearly always cause position or altitude maintenance problems. The graph in Figure 29b shows acceptable vibration levels that are consistently below 30 m/s^2^ and around 13 m/s^2^.

**Figure 30:**
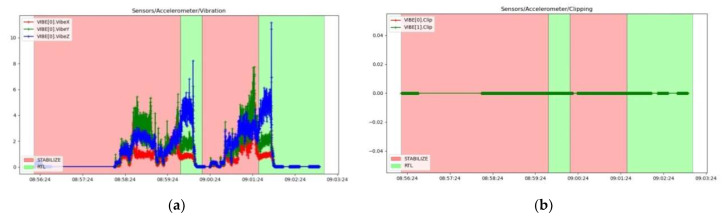
Vibration and clipping. (**a**) Accelerometer (0); (**b**) Clipping.

Similar results in terms of accelerometer vibrations (0) were found when performing another flight under similar conditions with the hexacopter, as shown in Figure 30a.

The phenomenon of accelerometer clipping, which means that the accelerometers have been exposed to a level of vibration that exceeds their full measurement range, is illustrated in Figure 30b. These are feedback signals to the control loop; therefore, if they are not operating in optimal parameters, the attitude control cannot be maintained. This phenomenon usually occurs when the drone collides with a hard object, such as crashing or landing on a hard surface. If the value increases during the flight, it is recommended to rebuild the damping system by fitting double adhesive strips or soft rubber mounts to allow the three-axis movement and avoid inducing vibration in the flight controller housing, which is then transmitted to the on-board sensors. In the case of the studied hexacopter platform, it was observed to have a value of 0. 

**Figure 31:**
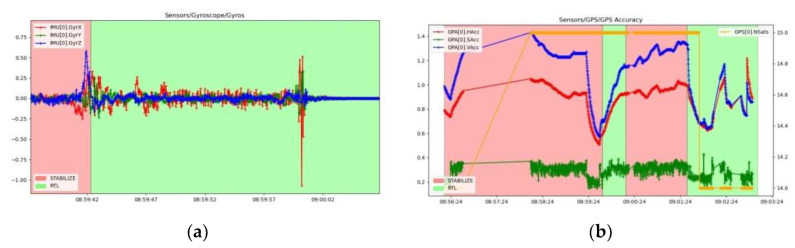
Gyro rotational speeds and accuracy of data received from GPS satellites. (**a**) Gyro rotational speeds in rad/s for IMU (0) and (1); (**b**) Accuracy of data received from GPS satellites.

To illustrate the operation of gyroscopes, in Figure 31a, the measured raw values of the rotational speeds in rad/s of the gyroscopes are represented. Very low values are recorded because the hexacopter does not pitch, roll or yaw during the ascent to the fixed-point hover altitude, but only compensates in very small increments the constant attitude. Because the controller has two IMUs, namely (0) and (1), both graphs are plotted comprising data from both subsystems. As expected, the measured values of both IMUs gyroscopes are identical, indicating the appropriate operation of these sensors. In the case of the GPS signal received by the GPS antenna, which has the Ublox M8N GPS built-in receiver, in Figure 31b, the accuracy of the received GPS signal is presented. HAcc indicates a horizontal positioning accuracy of 0.5–1.2 m, VAcc indicates a vertical positioning accuracy of 0.55–1.45 m and SAcc indicates a velocity measurement accuracy of up to 0.2–0.4 m/s. NSats indicates the number of satellites received, up to a maximum of 15.

While operating in one of the autonomous modes (loiter, RTL, auto, etc.), GPS position errors can cause the hexacopter to “feel” that it is in a different location than the correct one, which can lead to the drone flying aggressively to correct its perceived erroneous location information. These “errors” appear in both tlogs and dataflash logs as a decrease in the number of visible satellites and an increase in the horizontal HDop accuracy value.

**Figure 32:**
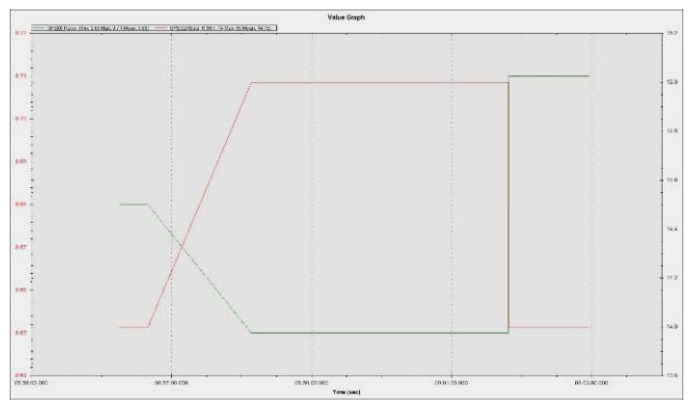
Accuracy of HDop positioning data received from GPS satellites.

Hdop values less than 1.5 are very good, and values above 2 could indicate that the GPS positions are not correct. Decreasing the number of satellites below 12 leads to erroneous measurements of the hexacopter position and speed relative to the ground. A significant change in these two values often accompanies a change in GPS position. Figure 32 proves that the number of received satellites is 15 and the horizontal position accuracy is 0.65–0.71 m, so both values correspond to a parameterized operation of the GPS signal receiving equipment from satellites.

**Figure 33:**
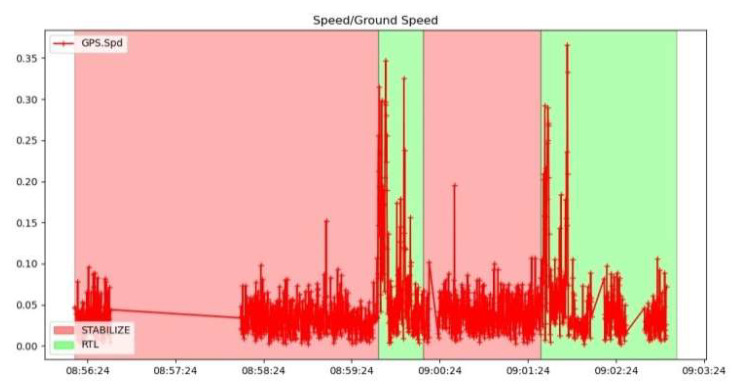
Relative speed of the drone to the ground.

Figure 33 shows a graph recording the relative speed of the hexacopter to the ground, based on the information received from the GPS. Given that the UAV performs the ascent and hover manoeuvre at a fixed point with small position adjustments, it can be seen that the value of this velocity is generally close to 0 m/s.

**Figure 34:**
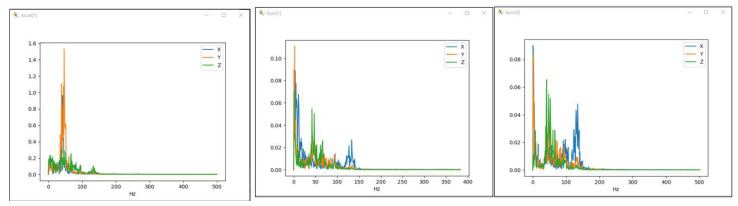
Vibration frequencies induced by motors rotation.

Mission planner, via the IMU batch sampler menu, has the option to record high-frequency data from IMU sensors to the flash data log on the flight controller. These data can be analysed after the flight to diagnose vibration-related problems using graphs created employing fast Fourier transforms (FFTs). A common feature of these plots is a peak at the “propeller blade crossing frequency”, that is, the frequency at which the blade passes over the arms and causes an acceleration in the frame. In the graphs presented in Figure 34 with data collected from accelerometers and gyroscopes, there are, however, certain noises corresponding to the eigenfrequencies of the motors. The accelerometer and gyroscope data show on the vertical axis the amplitude and on the horizontal axis the natural rotational frequency of the motors. The amplitude is not scaled to a useful value, so we cannot tell whether the levels of these values are high or low, which means that the graph is only useful for determining the frequency of vibrations. Vibrations at frequencies higher than 300 Hz can lead to attitude or position control problems. In this case, peak frequencies are observed at 40 Hz/2400 rpm, 47 Hz/2820 rpm, 95 Hz/5700 rpm, 130 Hz/7800 rpm and 153 Hz/9180 rpm.

It is possible to filter out these noises in order to increase the hexacopter performance and to allow a better parameter tuning by activating the harmonic notch filter(s). The harmonic notch filter is designed to match the frequency of the noise introduced by the engine rotation. Its value changes as the motor rotates by means of interpreting the value of the engine acceleration. The frequency is scaled up from the hover frequency and will never drop below this frequency. However, during a dynamic flight, it is quite common to reach low motor operating frequencies during propeller rotation. To address this, it is possible to modify the reference value in order to scale the filter to a lower frequency.

For the operation of the hexacopter beyond visual line of sight (BVLOS), the necessary components for implementation on the drone have been acquired. Ground and flight tests can be carried out to demonstrate their ability to control the hexacopter via 3G/4G LTE mobile networks. These include the Raspberry Pi 3B board, IR camera + EO camera and 4G LTE modem. Other hexacopter flights in different flight regimes, both manual and autonomous, can be carried out to test the drone limits. This research focused on the behaviour of the drone in stationary flight at a fixed point.

## 5. FEM Decision Support

The hexacopter was conceived to provide a robust structure for transporting large payloads. The aim of this approach is to ensure the stability of the hexacopter during stationary flight manoeuvres, to set out a composite simulation model for different analysis types and to validate the computational model in order to synchronize analytical, experimental and numerical results. FEM model reduction, efficiently tuning the discretization parameters with the available computational resources, was also a computational goal. 

The problem in the case of six propellers is that the rotating domains are close to each other, and the narrow space causes modelling and computational convergence issues. The results obtained from the FEM study were employed to optimize flight parameters, such as the rotor speed. Another important target of CFD simulations is the power requirements and the evaluation of the lift and drag forces. 

Hover flight is one of the most important flight regimes of the hexacopter, when the UAV requires maximum stability. In package delivery tasks, the fixed-point turbulences are essential, as they may act in the close proximity to buildings or even to the ground, especially in urban areas. This was considered when creating the flow domain around the hexacopter. The air pressure under the hexacopter is higher the closer the hexacopter is to the ground. It is therefore important to know the air pressure values so that the hexacopter remains stable. On the hexacopter frame, the pressure increases correspondingly as it approaches the ground or a target. Some of the turbulences are redirected on the drone components and on the rotors. The hexacopter can work in areas with dust, sand and even snow, which can then interact with the vehicle. This is another reason why the CFD study is essential to ensure the stability and safe operation of the hexacopter. 

### 5.1. CFD Approach

**Figure 35:**
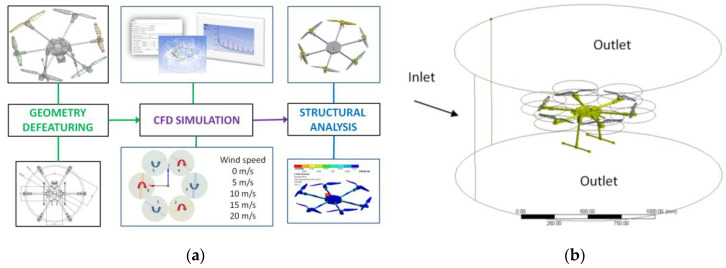
Fluid dynamics simulation. (**a**) CFD approach; (**b**) Enclosure.

**Figure 36:**
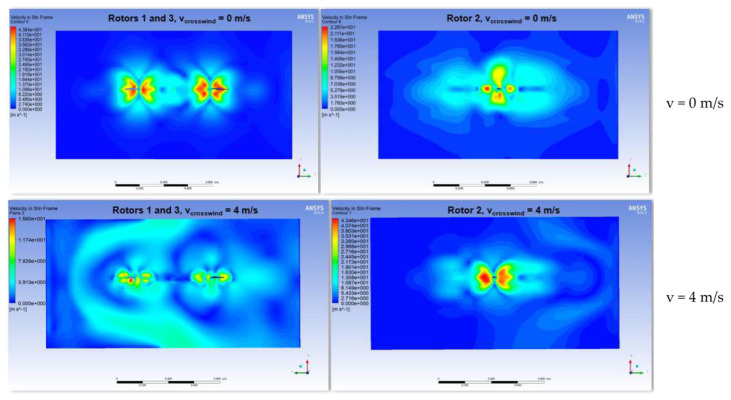

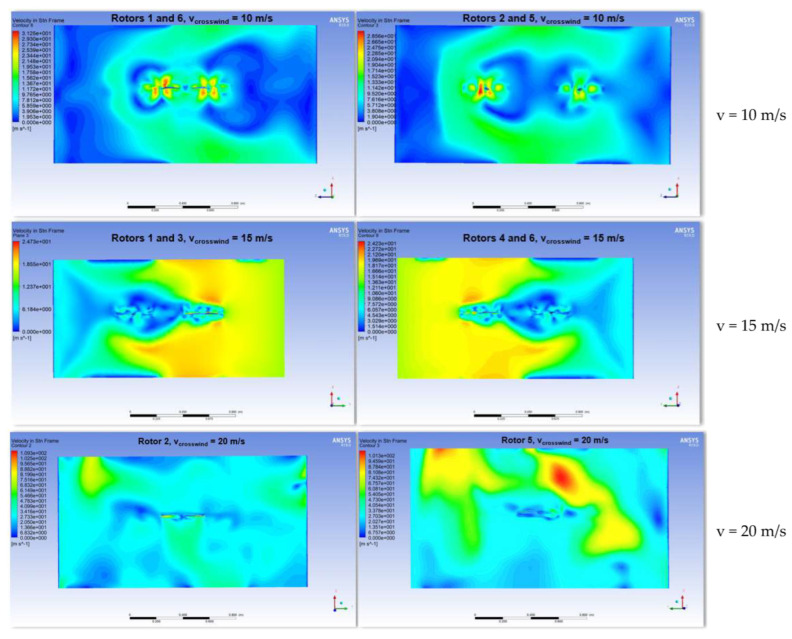
Velocity profile and dissipated turbulences for no wind and lateral wind scenarios.

The proposed study consists of three main steps: geometry modelling and defeaturing, followed by the CFD computations to extract the lift and drag forces on each propeller and then the evaluation of the displacements on the mechanical structure of the hexacopter for the worst-case scenario. The results were compared with the analytical ones. The workflow is represented in Figure 35a, encompassing the five environmental settings: lateral wind speed of 0 m/s, 4 m/s, 10 m/s, 15 m/s and 20 m/s (Figure 36a) and the CFD enclosure (Figure 35b). For all cases, the maximum rotational velocity of the propellers of 6500 rpm was considered. The final step is the fluid–structure interaction to evaluate the effect of the air fillets on the hexacopter structure.

The CFD simulation model explored a transient regime, and a corresponding mesh size was determined considering the Curant number. To avoid the numerical instability, this number must be 1 [87].

(1) $$Co=v_{max}\cdot \frac{dt}{dx}=1$$

where v_max_ is the maximum velocity in the flow domain, dt is the time increment and dx is the finite element size. 

The CFD model comprises nearly 2,900,000 elements with controlled inflation layers, named selections and multiple sizing options. The quality of the mesh was controlled, taking into account the skewness criterion, and the flow regime was based on the realizable k- ε flow model, employing advanced scalable wall functions and near-wall treatment interfaces.

In Figure 36, the contours of the velocities and pressures in the vertical plane are plotted, and the streamlines at rotor level for all the turbulent flows are processed. The influence of the wind on the fluid flow of the propellers occurring in the vertical plane can be observed, and the dissipation of the central turbulence is significant. The turbulence is deflected by the crosswind progressively for the cases v = 4 m/s, v = 10 m/s, v = 15 m/s and v = 20 m/s, respectively. The reaction thrust forces of the six propellers were between 0 and 38 N in absolute values. The lift forces were exported in a static analysis, and the spatial orientation of the hexacopter structure was evaluated as a function of the air pressure caused by the created turbulences, the rotational speed of the six rotors and the acceleration of the hexacopter. 

**Figure 37:**
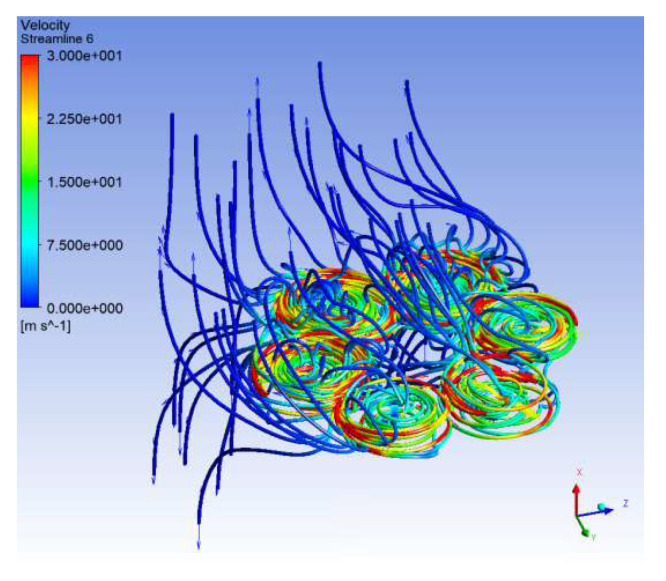
Streamlines and pressure contours on the rotors.

The velocity streamlines were processed in Figure 37, as well as the contour plot of the air pressure on the six drone rotors for the case of 10 m/s side wind speed. The maximum air pressure of 383 Pa on the rotors is low, but the influence on the hover flight attitude is further investigated in this paper in a fluid–structure interaction approach. The vortices and joint interferences at the small rotor spaces are compressed by the side wind, increasing the power consumption. The wind produces the movement of the coupled turbulences in the same direction, and this may cause the vibration of the rotors. The consequence is that a power increase and changes of the attack angle are necessary in order to maintain the desired hover flight. The aerodynamic efficiency is also ensured by high trust forces and small power consumption. These parameters were also reported by the CFD analysis.

**Figure 38:**
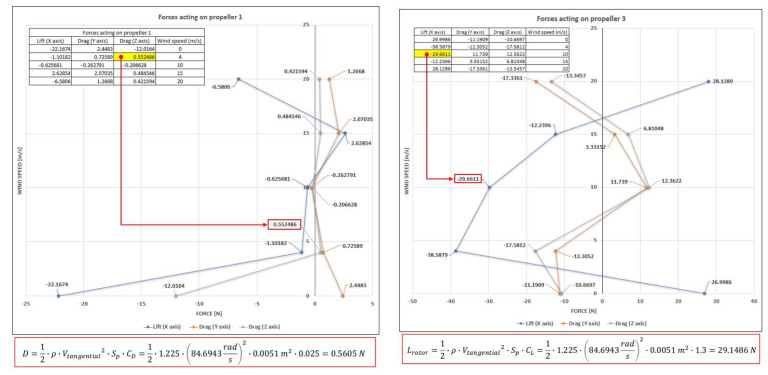
Drag and lift forces -numerical vs. analytic computation.

Figure 38 depicts the graph of the drag force on propeller 1 (0.552486 N) computed in the CFD simulation and the same force calculated analytically (0.5605 N). The difference is lower than 1.5%. The same comparison is performed for the lift force on propeller 3 (29.6611 N) as the output of the CFD simulation and by means of hand calculations (29.1486 N) employing the same analytical model. The difference remains low, of approximately 1.72% between the two methods. This validates the CFD computational model and the accuracy of the simulation results. The advantage of the results obtained in the virtual model is that they give access to information difficult to acquire by experiments in the entire CFD domain.

**Figure 39:**
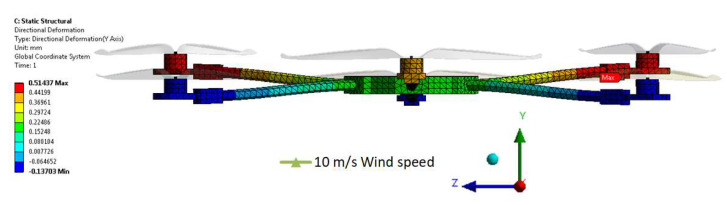
Maximum displacements on Y axis after 0.25s hover flight time.

Finally, a static analysis was completed, and the maximum displacement of the rotors and the frame have been processed in Figure 39. The static analysis on the hexacopter structural elements revealed that the directional deformation in the YZ plane caused by pressure distribution and the thrust forces resulting from the transient CFD computation at t = 0.25s in case of 10 m/s lateral wind is 0.51 mm. From this perspective, the structure is robust and does not influence the stability and the manoeuvrability of the hexacopter during hover.

Optimization of flight control parameters can be performed in respect to the CFD simulation results, namely, to the lift forces on the rotors (Z-axis), as well as the forces in the Z and Y directions, during hover. 

### 5.2. Dynamic Analysis and Hover Stability 

**Figure 40:**
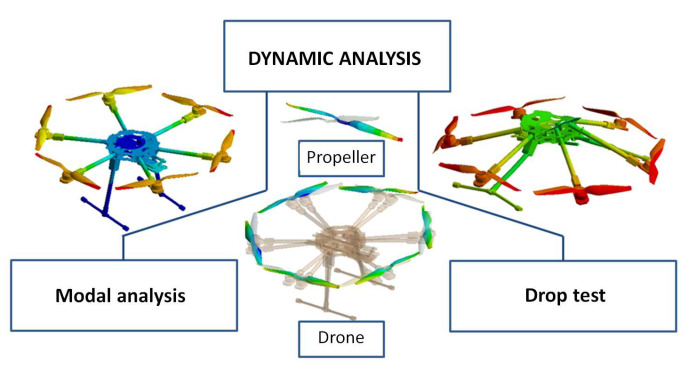
Hexacopter dynamic analysis using FEM.

The dynamic analysis focused on both the modal analysis and a drop test simulation to verify the stability and the structural integrity of the hexacopter at impact, as detailed in Figure 40.

The purpose of the modal analysis is to determine the eigenvalues for the drone’s structural elements and the propeller in order to design the hexacopter control system accordingly, to ensure the structural stability of the drone for the operational rotational speed of the propellers in surveillance, photography or recording operations. An orthotropic elastic epoxy carbon woven (230 GPa) prepreg material (Tsai-Wu) was employed for the hexacopter structural elements and the rotors. For reaching a realistic behaviour of the system, the mass of the assemblies and individual components of the hexacopter were carefully checked as recorded in Table 1.

**Figure 41:**
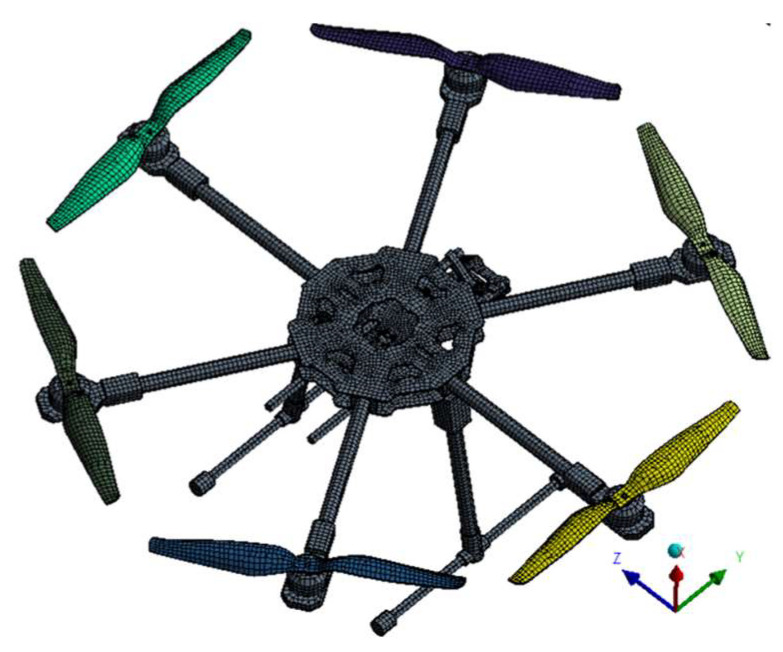
FEM model.

Figure 41 illustrates an efficient model in terms of computational time and mesh quality, based on shells and beams processed in the ANSA preprocessing system in order to combine different advanced discretization strategies. 

**Figure 42:**
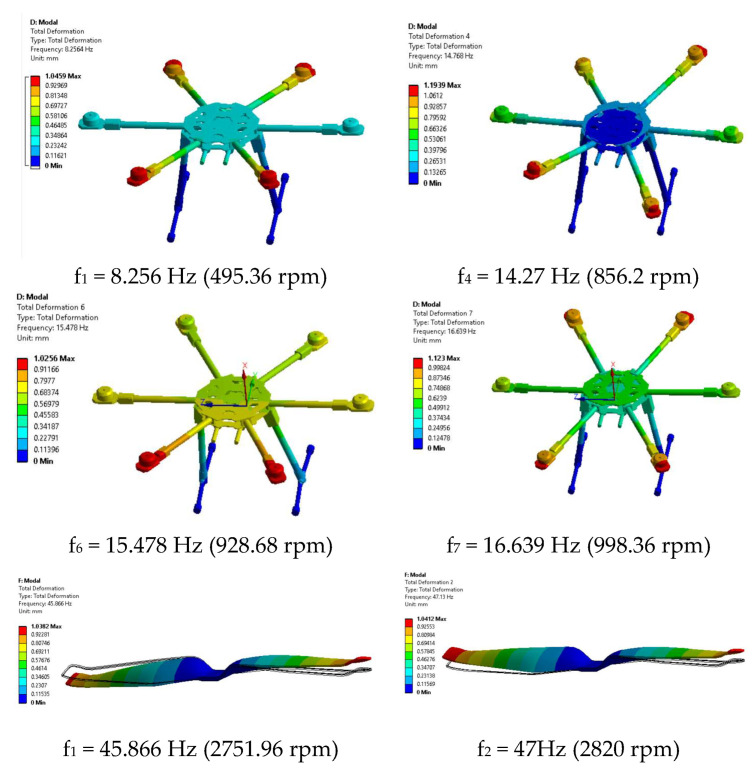
Mode shapes of the structural components and of the propeller.

The structure has two symmetry planes, and this is underlined in the modal response. In Figure 42, only relevant eigenvalues have been processed. Thus, the first mode shapes are bending modes in the vertical plane, followed by bending modes in the vertical plane combined with torsion in the horizontal plane. Participation factors were analysed to select the dominant vibration modes. For this purpose, ten mode shapes were calculated, with the normalization of the eigenvectors in respect to unity. The modal analysis of the hexacopter main structural components proves that there are no eigenfrequencies in the operational range; therefore, no problems arise from this point of view for controlling the attitude or position of the drone.

The study also confirms that the amplitude peak at 47 Hz (Figure 34, Section 5) corresponding to 2820 rpm is caused by the natural frequency of the propellers that are overlapping the manoeuvring frequency. This can lead to high stresses at the propeller hub and may cause them to exceed the material yield strength or simply reduces the fatigue strength of the rotors. Considering that the manoeuvring speed range [58.34 Hz/3500 rpm—66.67 Hz/4000 rpm] is at least 24% higher than the rotor eigenvalues, it can be assumed that no resonances can occur for the manoeuvring regime of the hexacopter, but only for the fixed-point stationary regime. The maximum rotor speeds for different flight conditions in the range [6500–8000 rpm] should be mindfully chosen, as resonances may occur on the third and fourth rotor eigenvalues, but the amplitudes are expected to be low.

The resonant rotational speeds have to be avoided and can be taken into account when monitoring the hover flight parameters. High-order frequencies may interfere with the flight parameters in manoeuvring mode, but this was not observed during the experiments. 

The hexacopter performance can also be improved by removing the resonances induced by the drives rotation from the operational range using dynamic harmonic notch filters. 

### 5.3. Hexacopter Drop Test 

**Figure 43:**
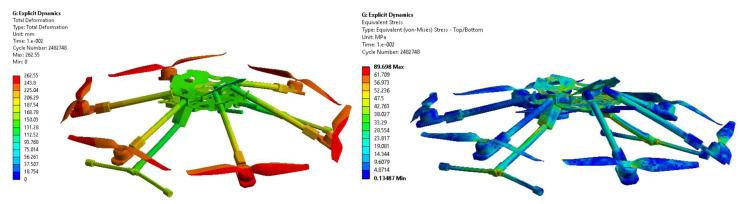
Drop test results at 0.1s after the impact.

The aim of this test in a virtual environment was to assess the possible damage of the hexacopter frame in the occurrence of a crash or an accidental landing on a stiff plate from a height of 20 m. All contacts between the components were considered rigid to avoid the mitigation effects. Because of the computationally intensive procedure, the simulation was stopped after the impact, before the complete kinetic energy consumption. Figure 43 depicts large displacements after the impact, but the stresses are not high due to the robust design. 

**Figure 44:**
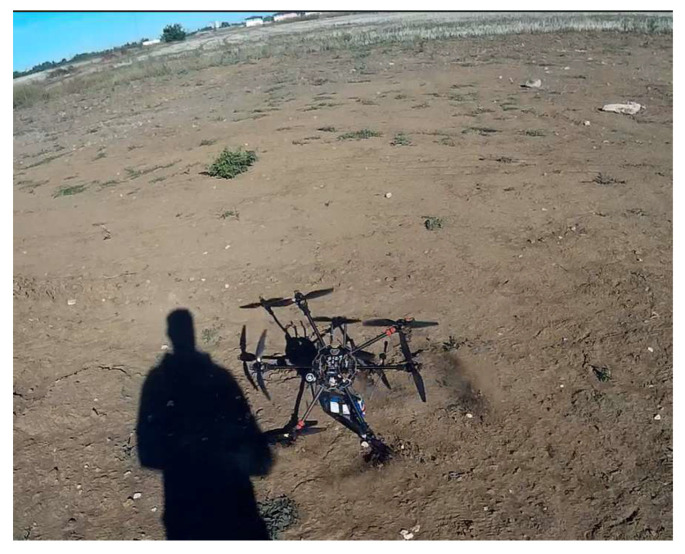
Hexacopter impact during field tests.

The fact that the postimpact stresses remain acceptable were reflected during the experiments, when the drone dropped somehow similarly. The hexacopter deforms strongly, but the original shape of the hexacopter can be recovered, even if large displacements can be observed. Experiments upheld the conclusions of the simulation test results when the hexacopter dropped from a lower height but on a less rigid soil (Figure 44). 

Impact simulations confirmed that when the hexacopter accidentally falls from a height of 20 m, the drone’s structure undergoes significant deformations, but no failure occurs. Maximum deformations arise on the vertical and horizontal struts in the joint areas. Due to the fact that the maximum strains are not high, the hexacopter structure can be recovered, as observed during field experiments. In this study, the hexacopter rigging was not considered; only the behaviour of the structural elements was taken into account. It was also observed that the rotors are much stiffer than in the solutions reported in the literature, confirming the robust design of the structural elements of the drone.

## 6. Conclusions and Future Work

This study was conducted employing two hexacopter variants in which the sensor system was analysed and synchronized so that the drone’s performance in stationary fixed-point flight was continuously improved. The flight parameters extracted from the tests were analysed, and the necessary corrective measures were taken accordingly to verify if the platform operates at optimal parameters, but also for stationary flight manoeuvres at hover, in average atmospheric conditions: temperature of 10–30°, 1–2 m/s wind and no precipitation.

The peculiarity of this work lies in the combination of experiments (both in the laboratory and in situ) with the FEM analysis in an original approach. The study also involved the development of a mathematical model for analytical calculation, not included in the paper, to determine the aerodynamic performance, as well as the verification of the solution of the hexacopter platform architecture. Only excerpts from the analytical computation were included in the discussion of the CFD simulation results for comparison purposes. Test outcomes were assessed, and conclusions regarding the numerical results were synchronized with the experimental ones. 

Regarding the hexacopter frame, it may be concluded that having a smaller distance between the rotors can improve the aerodynamic performances of the hexacopter by increasing the interference between the propellers. Similarly, the effects of interference between propellers are progressively reduced with the increase in rotor positioning. Thus, the homogeneity of the distribution of the airfoils and the shape and symmetry of the vortex are essential conditions for the hexacopter to generate better lift forces. These must be deeply understood in the sense that the simulation required to create flow domains may result in values slightly lower than the experimental, real values. 

FEM simulations are essential for achieving the aerodynamic stability and ensuring low power consumption and stable flight behaviour at high wind speeds, as well as achieving the ability of the vehicle to carry high payloads and increased operational areas of the hexacopter. These are mandatory requirements when launching a new, powerful hexacopter on the market. The simulation study can be continued by considering the hexacopter speed during manoeuvres and adjusting the rotational speeds of the propellers according to the experimental data.

The experiments completed employing the online platforms revealed a major drawback regarding the accuracy of the provided data. The deviation margin in respect to the real values was around ±15%; thus, when making the configurations and integrating the components, this error margin must be considered by choosing high-quality components and sensor systems to compensate this value. 

The data extracted from sensors mounted on the drone illustrated good results in terms of altitude, attitude, GPS, vibrations, response of the motors, temperature, atmospheric pressure and ESC readings, respectively. The hexacopter parameters acquired during hover flight allowed the remark regarding the accelerometers’ behaviour that they are not significantly affected by vibration during operation. Further adjustments to the current hexacopter-mounted sensors are underway to achieve even better results in terms of altitude and attitude estimation, positioning error compensation, engine control and command, telemetry data transmission, video signal transmission and radio interference compensation. Work regarding adding other sensors such as anemometers, LIDAR, acoustics and IR cameras is also in progress. Today, BVLOS is of major importance; thus, a variant of the BVLOS sensors kit is currently under development and will be mounted and tested on the hexacopter. 

## Figures and Tables

**Table 1:** Mass of the hexacopter components.

Hexacopter Component	Mass (kg)
Frame	0.833
Brushless electric motor	0.082
Electronic speed controller	0.026
13′′ Propeller	0.014
Avionics and accessories	0.763
12 Ah Battery 12 Ah	1.080

## Data Availability

Data described as result of this study are available on request to the corresponding author.

## Abbreviations

The following abbreviations are used in this manuscript:

AHRS	Attitude and heading reference system
AP	Autopilot
BLDC	Brushless DC electric motor
BVLOS	Beyond visual line-of-sight
CAD	Computer-aided design
CFD	Computational fluid dynamics
DRONE	Dynamic remotely operated navigation equipment
EKF	Extended Kalman filter
EO/IR	Electro-optical/infra-red
ESC	Electronic speed controller
FEM	Finite element method
FFT	Fast Fourier transforms
GIS	Geographic information system
GNSS	Global navigation satellite system
GPS	Global Positioning System
IMU	Inertial measurement unit
LIDAR	Light detection and ranging
MFD-LPTL	Multisensor fusion data analysis for low-power transmission lines
MOSFET	Metal–oxide–semiconductor field-effect transistor
PID	Proportional–integral–derivative
PPM	Pulse position modulation
PWM	Pulse width modulation
ROAV	Remotely operated air vehicle
ROS	Robotic operating system
RPAS	Remotely piloted aircraft system
SITL	Software-in-the-loop
SPSA	Simultaneous perturbation stochastic approximation
UAS	Unmanned aerial system
UAV	Unmanned aerial vehicle
UGV	Unmanned ground vehicle
UUV	Unmanned underwater vehicle
VTOL	Vertical takeoff and landing

## References

[1] Chu P.H., Huang Y.T., Pi C.H., Cheng S. (2022). Autonomous Landing System of a VTOL UAV on an Upward Docking Station Using Visual Servoing. IFAC-PapersOnLine.

[2] Sethi N., Ahlawat S. (2022). Low-fidelity design optimization and development of a VTOL swarm UAV with an open-source framework. Array.

[3] Patel T., Kumar M., Abdallah S. (2022). Control of Hybrid Transitioning Morphing-wing VTOL UAV. IFAC-PapersOnLine.

[4] Bahari M., Rostami M., Entezari A., Ghahremani S., Etminan M. (2022). A comparative analysis and optimization of two supersonic hybrid SOFC and turbine-less jet engine propulsion system for UAV. Fuel.

[5] Bahari M., Rostami M., Entezari A., Ghahremani S., Etminan M. (2022). Performance evaluation and multi-objective optimization of a novel UAV propulsion system based on PEM fuel cell. Fuel.

[6] Zhou K., Liu Z., Zhang X., Liu H., Meng N., Huang J., Qi M., Song X., Yan X. (2022). A kW-level integrated propulsion system for UAV powered by PEMFC with inclined cathode flow structure design. Appl. Energy.

[7] Lu S.H., Kuo R.J., Ho Y.T., Nguyen A.T. (2022). Improving the efficiency of last-mile delivery with the flexible drones traveling salesman problem. Expert Syst. Appl..

[8] Jung H., Kim J. (2022). Drone scheduling model for delivering small parcels to remote islands considering wind direction and speed. Comput. Ind. Eng..

[9] Jeong H.Y., Song B.D., Lee S. (2022). Optimal scheduling and quantitative analysis for multi-flying warehouse scheduling problem: Amazon airborne fulfillment center. Transp. Res. Part C Emerg. Technol..

[10] Zhai D., Wang C., Cao H., Garg S., Hassan M.M., Al Qahtani S.A. (2022). Deep neural network-based UAV deployment and dynamic power control for 6G-Envisioned intelligent warehouse logistics system. Future Gener. Comput. Syst..

[11] Mourtzis D., Angelopoulos J., Panopoulos N. (2021). UAVs for Industrial Applications: Identifying Challenges and Opportunities from the Implementation Point of View. Procedia Manuf..

[12] Yuan C.S., Cheng W.H., Su S.Y., Chen W.H. (2021). Field measurement of spatiotemporal distributions of ambient concentrations of volatile organic compounds around a high-tech industrial park using a drone. Atmos. Pollut. Res..

[13] Wang Y., Li Y., Yin F., Wang W., Sun H., Li J., Zhang K. (2022). An intelligent UAV path planning optimization method for monitoring the risk of unattended offshore oil platforms. Process Saf. Environ. Prot..

[14] Cho J., Lim G., Biobaku T., Kim S., Parsaei H. (2015). Safety and Security Management with Unmanned Aerial Vehicle (UAV) in Oil and Gas Industry. Procedia Manuf..

[15] Garcia-Vasquez A.C., Mokari E., Samani Z., Fernald A. (2022). Using UAV-thermal imaging to calculate crop water use and irrigation efficiency in a flood-irrigated pecan orchard. Agric. Water Manag..

[16] Cheng K.H., Jiao J.J., Luo X., Yu S. (2022). Effective coastal Escherichia coli monitoring by unmanned aerial vehicles (UAV) thermal infrared images. Water Res..

[17] Qin G., Xu Y., Li F., Zhou W., Li W., Zhao G. (2022). Calibration of an airborne γ-ray spectrometer based on an unmanned aerial vehicle using a point source. Ann. Nucl. Energy.

[18] Lipovský P., Novotnák J., Blažek J. (2022). Possible Utilization of Low Frequency Magnetic Fields in Short Range Multirotor UAV Detection System. Transp. Res. Procedia.

[19] Amodu O.A., Busari S.A., Othman M. (2022). Physical layer aspects of terahertz-enabled UAV communications: Challenges and opportunities. Veh. Commun..

[20] Xie C., Yang C. (2020). A review on plant high-throughput phenotyping traits using UAV-based sensors. Comput. Electron. Agric..

[21] Da Silva S.D.P., Eugenio F.C., Fantinel R.A., Amaral L.D.P., dos Santos A.R., Mallmann C.L., dos Santos F.D., Pereira R.S., Ruoso R. (2023). Modeling and detection of invasive trees using UAV image and machine learning in a subtropical forest in Brazil. Ecol. Inform..

[22] Amarasingam N., Ashan Salgadoe A.S., Powell K., Gonzalez L.F., Natarajan S. (2022). A review of UAV platforms, sensors, and applications for monitoring of sugarcane crops. Remote Sens. Appl. Soc. Environ..

[23] Hao Z., Li M., Yang W., Li X. (2022). Evaluation of UAV spraying quality based on 1D-CNN model and wireless multi-sensors system. Inf. Process. Agric..

[24] Lin B., Xu J., Yin C., Chen L., You Y., Hu L. (2022). An ultralight dual-wavelength and dual-beam chemical sensor on small UAV for in-situ determination of phosphate and nitrite in surface water. Sens. Actuators B Chem..

[25] Mumuni F., Mumuni A., Amuzuvi C.K. (2022). Deep learning of monocular depth, optical flow and ego-motion with geometric guidance for UAV navigation in dynamic environments. Mach. Learn. Appl..

[26] Bauer P., Kun S. (2022). Optical flow-based angular rate sensor fault detection on UAVs. IFAC-PapersOnLine.

[27] Stöcker C., Bennett R., Koeva M., Nex F., Zevenbergen J. (2022). Scaling up UAVs for land administration: Towards the plateau of productivity. Land Use Policy.

[28] Wang T., Mei X., Alex Thomasson J., Yang C., Han X., Yadav P.K., Shi Y. (2022). GIS-based volunteer cotton habitat prediction and plant-level detection with UAV remote sensing. Comput. Electron. Agric..

[29] Tan Y., Li G., Cai R., Ma J., Wang M. (2022). Mapping and modelling defect data from UAV captured images to BIM for building external wall inspection. Autom. Constr..

[30] Yap Y.L., Toh W., Giam A., Yong F.R., Chan K.I., Tay J.W.S., Teong S.S., Lin R., Ng T.Y. (2023). Topology optimization and 3D printing of micro-drone: Numerical design with experimental testing. Int. J. Mech. Sci..

[31] Tolba M., Shirinzadeh B. (2022). Generic modeling and control of unbalanced multirotor UAVs. Aerosp. Sci. Technol..

[32] Lee S., Chung W., Son H. (2022). Online parameter identification framework for a multirotor UAV: Application to an arm stretchable morphing multirotor. Mech. Syst. Signal Process..

[33] Michel N., Wei P., Kong Z., Sinha A.K., Lin X. (2022). Modeling and validation of electric multirotor unmanned aerial vehicle system energy dynamics. eTransportation.

[34] Lim D., Kim H., Yee K. (2022). Uncertainty propagation in flight performance of multirotor with parametric and model uncertainties. Aerosp. Sci. Technol..

[35] Zhang H., Qi L., Wan J., Musiu E.M., Zhou J., Lu Z., Wang P. (2022). Numerical simulation of downwash airflow distribution inside tree canopies of an apple orchard from a multirotor unmanned aerial vehicle (UAV) sprayer. Comput. Electron. Agric..

[36] Liu Z., Zhang Y., Chen H., Zhang Z. (2022). Incremental control system design and flight tests of a micro-coaxial rotor UAV. Aerosp. Sci. Technol..

[37] Mishra A., Pal S., Singh P. (2022). Design and analysis of an Eight Rotor Co-Axial UAV using carbon fiber composites. Mater. Today: Proc..

[38] Liscouët J., Pollet F., Jézégou J., Budinger M., Delbecq S., Moschetta J.M. (2022). A methodology to integrate reliability into the conceptual design of safety-critical multirotor unmanned aerial vehicles. Aerosp. Sci. Technol..

[39] Darvishpoor S., Roshanian J., Raissi A., Hassanalian M. (2022). Configurations, flight mechanisms, and applications of unmanned aerial systems: A review. Prog. Aerosp. Sci..

[40] Delbecq S., Budinger M., Ochotorena A., Reysset A., Defay F. (2020). Efficient Sizing and Optimization of Multirotor Drones Based on Scaling Laws and Similarity Models. Aerosp. Sci. Technol..

[41] Gupta A.K., Jha V., Gupta V.K. (2014). Design and Development of Remote Controlled Autonomous Synchronic Hexarotor Aerial (ASHA) Robot. Procedia Technol..

[42] Suprapto B.Y., Heryanto A., Suprijono H., Muliadi J., Kusumoputro B. Design and Development of Heavy-lift Hexacopter for Heavy Payload. Proceedings of the International Seminar on Application for Technology of Information and Communication (iSemantic).

[43] Setiono F.Y., Candrasaputra A., Prasetyo T.B., Santoso K.L.B. Designing and Implementation of Autonomous Hexacopter as Unmanned Aerial Vehicle. Proceedings of the 8th International Conference on Information Technology and Electrical Engineering (ICITEE).

[44] Verbeke J., Hulens D., Ramon H., Goedemé T., de Schutter J. The Design and Construction of a High Endurance Hexacopter suited for Narrow Corridors. Proceedings of the International Conference on Unmanned Aircraft Systems (ICUAS).

[45] Abarca M., Saito C., Angulo A., Paredes J.A., Cuellar F. Design and Development of an Hexacopter for Air Quality Monitoring at High Altitudes. Proceedings of the 13th IEEE Conference on Automation Science and Engineering (CASE), Xi’an.

[46] Arellano-Quintana V.M., Portilla-Flores E.A., Merchan-Cruz E.A., Nino-Suarez P.A. Multirotor Design Optimization Using a Genetic Algorithm. Proceedings of the International Conference on Unmanned Aircraft Systems (ICUAS).

[47] Ferrarese G., Giulietti F., Avanzini G. (2013). Modeling and Simulation of a Quad-Tilt Rotor Aircraft. IFAC Proc. Vol..

[48] Ryll M., Bicego D., Franchi A. Modeling and Control of FAST-Hex: A Fully–Actuated by Synchronized–Tilting Hexacopter. Proceedings of the IEEE/RSJ International Conference on Intelligent Robots and Systems (IROS).

[49] Tadokoro Y., Ibuki T., Sampei M. (2017). Maneuverability Analysis of a Fully-Actuated Hexrotor UAV Considering Tilt Angles and Arrangement of Rotors. IFAC PapersOnLine.

[50] Rajappa S., Ryll M., Bulthoff H.H., Franchi A. Modeling, Control and Design Optimization for a Fully actuated Hexacopter Aerial Vehicle. Proceedings of the IEEE International Conference on Robotics and Automation (ICRA).

[51] Köse O., Oktay T. (2022). Hexarotor Yaw Flight Control with SPSA PID Algorithm and Morphing. Int. J. Intell. Syst. Appl. Eng..

[52] Mehmood H., Nakamura T., Johnson E.N. A Maneuverability Analysis of a Novel Hexacopter UAV Concept. Proceedings of the International Conference on Unmanned Aircraft Systems (ICUAS).

[53] Budinger M., Reysset A., Ochotorena A., Delbecq S. (2020). Scaling laws and similarity models for the preliminary design of multirotor drones. Aerosp. Sci. Technol..

[54] Hussein M., Nouacer R. (2022). Reference architecture specification for drone systems. Microprocess. Microsyst..

[55] Cao S., Fan Q., Yu W.J., Wang L.T., Ni S., Chen J. (2022). Multi-Sensor fusion and data analysis for operating conditions of low power transmission lines. Measurement.

[56] Severin T., Soffker D. (2022). Sensor optimization for altitude estimation of spraying drones in vineyards. IFAC-PapersOnLine.

[57] Pena P.F., Ragab A.R., Luna M.A., Isaac M.S.A., Campoy P. (2022). WILD HOPPER: A heavy-duty UAV for day and night firefighting operations. Heliyon.

[58] Ravin K., Agrawal A.K. (2021). Drone GPS data analysis for flight path reconstruction: A study on DJI, Parrot & Yuneec make drones. Forensic Sci. Int. Digit. Investig..

[59] Sree Ezhil V.R., Rangesh Sriram B.S., Christopher Vijay R., Yeshwant S., Sabareesh R.K., Dakkshesh G., Raffik R. (2022). Investigation on PID controller usage on Unmanned Aerial Vehicle for stability control. Mater. Today: Proc..

[60] Madokoro H., Kiguchi O., Nagayoshi T., Chiba T., Inoue M., Chiyonobu S., Nix S., Woo H., Sato K. (2021). Development of Drone-Mounted Multiple Sensing System with Advanced Mobility for In Situ Atmospheric Measurement: A Case Study Focusing on PM2.5. Local Distribution, Sensors.

[61] Megayanti M., Nugraha Y.P., Sary I.P., Hidayat E., Trilaksono B.R. Modeling and Implementation of Hexacopter Guidance System Using Fuzzy Logic Control Under Wind Disturbance. Proceedings of the IEEE 8th International Conference on System Engineering and Technology (ICSET).

[62] Sharipov D., Abdullaev Z., Tazhiev Z., Khafizov O. Implementation of a mathematical model of a hexacopter control system. Proceedings of the International Conference on Information Science and Communications Technologies (ICISCT).

[63] Toledo J., Acosta L., Perea D., Morales N. (2015). Stability and performance analysis of unmanned aerial vehicles: Quadcopter against Hexrotor. IET Control Theory Appl..

[64] Wen F.-H., Hsiao F.-Y., Shiau J.-K. (2021). Analysis and Management of Motor Failures of Hexacopter in Hover. Actuators.

[65] Leishman R., Macdonald J., McLain T., Beard R. Relative Navigation and Control of a Hexacopter. Proceedings of the IEEE International Conference on Robotics and Automation.

[66] Derawi D., Salim N.D., Azizi M., Rahman A., Mazlan S.A., Zamzuri H. Modeling, Attitude Estimation, and Control of Hexacopter Micro Aerial Vehicle (MAV). Proceedings of the IEEE International Conference on Industrial Technology (ICIT).

[67] Derawi D., Salim N.D., Zamzuri H., Liu H., Azizi M., Rahman A., Mazlan S.A. Robust Attitude Controler for Uncertain Hexacopter Micro Aerial Vehicles (MAVs). Proceedings of the IEEE/RSJ International Conference on Intelligent Robots and Systems (IROS).

[68] Poksawat P., Wang L. Automatic Tuning of Hexacopter Attitude Control Systems with Experimental Validation. Proceedings of the 21st International Conference on System Theory, Control and Computing (ICSTCC).

[69] Zheng Y., Dong L., Wang Q. Multi-Rotor UAV Attitude Calculation Based on Extended Kalman Filter. Proceedings of the 30th Chinese Control and Decision Conference (CCDC).

[70] Benzemrane K., Damm G., Santosuosso G.L. Adaptive Observer and Kalman Filtering. Proceedings of the 17th World Congress, The International Federation of Automatic Control.

[71] Benzerrouk H., Nebylov A., Salhi H. (2016). Quadcopter UAV state estimation based on High-Degree Cubature Kalman filter. IFAC-PapersOnLine.

[72] Neumann P.P., Bartholmai M. (2015). Real-time wind estimation on a micro unmanned aerial vehicle using its inertial measurement unit. Sens. Actuators.

[73] Sushchenko O.A., Beliavtsev Y.V. Modelling of Inertial Sensors in UAV Systems. Proceedings of the IEEE 4th International Conference Actual Problems of Unmanned Aerial Vehicles Developments (APUAVD).

[74] Heise C.D., Falconi G.P., Holzapfel F. Hexacopter Outdoor Flight Test Results of an Extended State Observer based Controller. Proceedings of the IEEE International Conference on Aerospace Electronics and Remote Sensing Technology (ICARES).

[75] Dong W., Gu G.Y., Zhu X., Ding H. (2014). High-performance trajectory tracking control of a quadcopter with disturbance observer. Sens. Actuators.

[76] Lee S.J., Kim S., Johansson K.H., Kim H.J. Robust Acceleration Control of a Hexacopter UAV with a Disturbance Observer. Proceedings of the IEEE 55th Conference on Decision and Control (CDC).

[77] Seah C.H., Inyang I.J., Whidborne J.F. (2017). Bilinear Modelling and Attitude Control of a quadcopter. IFAC PapersOnLine.

[78] Herrada F.J., García-Martínez J., Fraile A., Hermanns L.K.H., Montáns F.J. (2017). A method for performing efficient parametric dynamic analyses in large finite element models undergoing structural modifications. Eng. Struct..

[79] Karthik Vinayaga K., Vasanthanathan A., Nagaraj P. (2018). Finite element modeling of smart piezoelectric beam using ANSYS. Mater. Today Proc..

[80] Ryzhakov P., Rossi R., Viña A., Oñate E. (2013). Modelling and simulation of the sea-landing of aerial vehicles using the Particle Finite Element Method. Ocean. Eng..

[81] Jiapeng T., Ping X., Baoyuan Z., Bifu H. (2013). A finite element parametric modeling technique of aircraft wing structures. Chin. J. Aeronaut..

[82] Papa U., Russo S., Lamboglia A., Del Core G., Iannuzzo G. (2017). Health structure monitoring for the design of an innovative UAS fixed wing through inverse finite element method (iFEM). Aerosp. Sci. Technol..

[83] Felismina R., Silva M., Mateus A., Malça C. (2017). Study on the aerodynamic behavior of a UAV with an applied seeder for agricultural practices. AIP Conf. Proc..

[84] Lei Y., Cheng M. (2020). Aerodynamic performance of a Hex-rotor unmanned aerial vehicle with different rotor spacing. Meas. Control..

[85] Lei Y., Cheng M. (2019). Aerodynamic Performance of Hex-Rotor UAV Considering the Horizontal Airflow. Appl. Sci..

[86] Zheng Y., Yang S., Liu X., Wang J., Norton T., Chen J., Tan Y. (2018). The computational fluid dynamic modeling of downwash flow field for a six-rotor UAV. Front. Agric. Sci. Eng..

[87] Courant R., Friedrichs K., Lewy H. (1967). On the partial difference equations of mathematical physics. IBM J. Res. Dev..

